# A Systematic Review of the Validity and Reliability of Assessment Tools for Executive Function and Adaptive Function Following Brain Pathology among Children and Adolescents in Low- and Middle-Income Countries

**DOI:** 10.1007/s11065-022-09538-3

**Published:** 2022-03-29

**Authors:** Kwabena Kusi-Mensah, Nana Dansoah Nuamah, Stephen Wemakor, Joel Agorinya, Ramata Seidu, Charles Martyn-Dickens, Andrew Bateman

**Affiliations:** 1 Department of Psychiatry, University of Cambridge, Clifford Allbutt Building, Cambridge Biomedical Campus CB2 OAH, Cambridge, UK; 2 Komfo Anokye Teaching Hospital, P. O. Box 1934, Kumasi, Ghana; 3 Pantang Hospital, Accra, Ghana; 4 Department of Psychiatry, University of Michigan Health System, 1500 E Medical Center Dr, Ann Arbor, MI 48109 USA; 5 Accra Psychiatric Hospital, Accra, Ghana; 6 School of Health and Social Care, University of Essex, Colchester, UK

**Keywords:** Executive Function, Adaptive function, Assessment instruments, Systematic review, Psychometrics, Developing countries, Children

## Abstract

Minimal but increasing number of assessment instruments for Executive functions (EFs) and adaptive functioning (AF) have either been developed for or adapted and validated for use among children in low and middle income countries (LAMICs). However, the suitability of these tools for this context is unclear. A systematic review of such instruments was thus undertaken. The Systematic review was conducted following the Preferred Reporting Items for Systematic Review and Meta-Analysis (PRISMA) checklist (Liberati et al., in *BMJ (Clinical Research Ed.), 339,* 2009). A search was made for primary research papers reporting psychometric properties for development or adaptation of either EF or AF tools among children in LAMICs, with no date or language restrictions. 14 bibliographic databases were searched, including grey literature. Risk of bias assessment was done following the COSMIN (**CO**nsensus-based **S**tandards for the selection of health status **M**easurement **IN**struments) guidelines (Mokkink et al., in *Quality of Life Research,* *63*, 32, 2014). For EF, the Behaviour Rating Inventory of Executive Functioning (BRIEF- multiple versions), Wisconsin Card Sorting Test (WCST), Go/No-go and the Rey-Osterrieth complex figure (ROCF) were the most rigorously validated. For AFs, the Vineland Adaptive Behaviour Scales (VABS- multiple versions) and the Child Function Impairment Rating Scale (CFIRS- first edition) were most validated. Most of these tools showed adequate internal consistency and structural validity. However, none of these tools showed acceptable quality of evidence for sufficient psychometric properties across all the measured domains, particularly so for content validity and cross-cultural validity in LAMICs. There is a great need for adequate adaptation of the most popular EF and AF instruments, or alternatively the development of purpose-made instruments for assessing children in LAMICs.

**Systematic Review Registration numbers:** CRD42020202190 (EF tools systematic review) and CRD42020203968 (AF tools systematic review) registered on PROSPERO website (https://www.crd.york.ac.uk/prospero/).

**Supplementary Information:**

The online version contains supplementary material available at 10.1007/s11065-022-09538-3.

## Introduction

### Rationale

Only 10% of research into child and adolescent mental health (CAMH) problems is carried out in low-and-middle-income countries (LAMICs) (Kessler et al., 2007; Merikangas et al., 2009). Meanwhile, over 90% of the world’s children live in LAMICs (WHO, 2008) and this proportion is only set to grow with the high birth rate in these countries. Historically the focus of public health in LAMICs has been on communicable diseases like HIV and Malaria. However, as the standard of living of LAMICs continues to improve, the data shows that in recent years the gradual decrease in infant mortality has resulted in an increasing shift toward non-communicable diseases such as neurodevelopmental disorders (NDDs) (Bakare et al., 2014) and CAMH disorders, which impact cognitive function. Children who would otherwise have died from various infections and birth injuries are now surviving, but surviving with the sequalae of the varied insults suffered at birth and the perinatal period, which are common in LAMICs because of poor obstetric care (Omigbodun & Bella, 2004). The common pathway of many of these conditions are neurobehavioral difficulties often described as Acquired Brain Injury (Bennett et al., 2005; Stuss, 1983) which affects brain function and the mental health and well-being of these children. It is within this context that accurate assessment of executive dysfunction and adaptive functioning, as known sequalae for brain injury, becomes quite important for children in LAMICs.

Executive functions (EF) may be defined as “top-down control processes” of human behaviour (Diamond, 2013) whose primary function is “supervisory control” (Stuss & Alexander, 2000) and includes such abilities as initiation, planning, and decision-making (Diamond, 2013). Better EF is linked to many positive outcomes (Diamond, 2013) such as greater success in school (Duncan et al., 2007; St Clair-Thompson & Gathercole, 2006), while deficits in EF are associated with slow school progress (Morgan et al., 2017) difficulties in peer relationships (Tseng & Gau, 2013) and poor employment prospects (Bailey, 2007). This may be because EF’s have also been presented as potential endophenotypes of various childhood mental disorders such as Hyperkinetic Disorder (ICD 10^1^ code: F90.1; also commonly called Attention Deficit Hyperactivity Disorder (ADHD)) (Doyle et al., 2005). Behaviourally, EF deficits may manifest as distractibility, fidgetiness, poor concentration, chaotic organization of materials, and trouble completing work (Bathelt et al., 2018). Given the difficulties seen, it is therefore important that mental health and rehabilitation services are able to pinpoint areas of greatest difficulty and target interventions appropriately and cost effectively through accurate assessments (Simblett et al., 2012).

Adaptive functioning on the other hand is an area that is just beginning to be examined (Semrud-Clikeman et al., 2017). It is defined as behaviours necessary for age-appropriate, independent functioning in social, communication, daily living or motor areas (Matson et al., 2009), tapping into the ability to carry out everyday tasks within age and context appropriate constraints (World Health Organization, 2001). In this present study, the term was restricted to the narrow scope of adaptive function following brain injury/brain pathology.

Adaptive functioning may be viewed as the practical expression of executive functions in an everyday functional context. Executive function abilities are related and have predictive power over adaptive behaviour (Clark et al., 2002) in both typical and atypical populations according several studies (Gardiner & Iarocci, 2018; Gilotty et al., 2010; Gligorović & Ðurović, 2014; Low Kapalu et al., 2018; Perna et al., 2012; Pugliese et al., 2015; Sabat et al., 2020; Schonfeld et al., 2007; Ware et al., 2012; Zorza et al., 2016). Specific domains of adaptive behaviours and academic achievement may, in part, depend on executive function capacities (Clark et al., 2002). Specifically, the core domains of EF- working memory, inhibition, and cognitive flexibility- have been shown to relate to the domains of adaptive behaviour as conceptual skills (e.g., language and the understanding of time, money, and number concepts) (Gilotty et al., 2010; Pugliese et al., 2015; Sabat et al., 2020) and practical skills (e.g., personal care, occupational and safety capabilities, use of money and transportation, and following of schedules and routines) (Perna et al., 2012; Sabat et al., 2020) and social skills (Gilotty et al., 2010; Pugliese et al., 2015; Zorza et al., 2016). Therefore, reviewing tools for the two related constructs seemed like an appropriate approach to take. Therefore, assessed together, EF and AF could provide the most utility to LMIC clinicians, depending on whether the goal is to focus interventions from a specific domains’ perspective or specific areas of functional deficit in a day-to-day context for interventions.

At this point, one may wonder why of all the cognitive functioning constructs we chose to review tools assessing executive functions and adaptive functioning, and not say IQ. We wanted to review constructs that found the widest applicability trans-diagnostically and the most utility in the clinical setting, and for which interventions could be most directly designed. The information that assessing other psychological constructs related to frontal lobe functioning such as IQ might give, may not be as actionable as what a comprehensive assessment of the domains of EF and AF would provide. For example, an IQ assessment may be useful in diagnosing intellectual disability and in school placement (which are both very important of course), whereas an assessment of EF and AF could lead to identifying domains and areas of functional deficit in the child’s life (across several diagnostic labels) that would lend itself most immediately to therapeutic interventions. Furthermore, AF assessment would also allow clinicians to determine the level of support required (Association & Association, 2013). Finally, even in the developed world with the full range of neuropsychological services available, neuropsychological assessments such as EF and AF assessments alone represents a significant proportion of all assessment services by clinical psychologists (up to 21% according to a large national representative survey among US psychologists (Camara et al., 2000). It therefore seemed more useful for resource constrained LMICs to do a systematic review of such tools for EF and AF than for any other psychological construct at the present time.

Several tools have been developed to assess executive and adaptive function in Western or High-Income-Country (HIC) populations, which perpetuates the trend of skewing research towards wealthier countries as noted above (Kessler et al., 2007; Merikangas et al., 2009). However, not much is known about the nature and quality of tools developed for LAMIC populations. A recent scoping review of EF tools among adolescents was limited in scope, and did not focus particularly on LAMICs (Nyongesa et al., 2019). More importantly, this study did not evaluate risk of bias of the eligible papers (particularly noteworthy was the lack of focus on assessing risk of bias of content validity and cross cultural validity) but only reported on the results declared therein (Nyongesa et al., 2019). Another recent scoping review- this time focused on NDD’s among children in LAMICs also reported regarding EF and AF tools used in LAMICs that, only a few tests have been used to assess executive function in children in LAMICs (Semrud-Clikeman et al., 2017). This paper also failed to do a critical appraisal, though that was outside its scope.

There is a high burden of the known causes of brain injury in developing countries (Bitta et al., 2017; Merikangas et al., 2009) and therefore a rigorous critical appraisal of appropriate assessment tools for EF and AF in this specific context will be highly desirable. Towards this goal of elucidating the issue of assessment of executive and adaptive functioning among children in the context of LAMICs, a scoping review of the subject was undertaken by the authors in an earlier paper to broadly map out the kinds of instruments that had either been newly developed or adapted for use in this context, the results of which are reported elsewhere (Kusi-Mensah et al., 2021). However, reviewing the quality of evidence found in the reviewed papers was beyond the scope of that scoping review. This present paper, therefore, is a continuation of that project, seeking to critically appraise the quality of evidence for the results of the scoping review, and to thus make more definitive recommendations on the best instruments with the most high-quality evidence for use among children in LAMICs.

### Objectives

The present study seeks to undertake a systematic review of published literature on the reliability and validity of assessment tools for executive functioning and adaptive functioning among children in LAMIC contexts. The purpose of this is to critically appraise and summarise the evidence for the scientific rigour of the methodologies used (risk of bias), and the results presented (psychometric measurement properties established) for the tools which have been developed, adapted or validated among children in developing country contexts, as well as document any knowledge gaps that may exist.

The following research questions were therefore formulated:

1. What is the quality of adaptation (including content validation) of existing HIC-derived assessment tools for executive functioning or adaptive functioning among children in LAMICs?
2. What is the nature and quality of evidence undergirding newly developed and purpose-made tools for assessment of executive functioning or adaptive functioning among children in LAMICs?
3. What is the nature and quality of evidence supporting the psychometric properties (validity and reliability) of all HIC-derived assessment tools for executive functioning or adaptive functioning among children in LAMICs?

## Methods

### Protocol and Registration

This systematic review was conducted following the Preferred Reporting Items for Systematic Review and Meta-Analysis (PRISMA) checklist (Liberati et al., 2009), and our protocol was written according to the PRISMA- Protocol extension (PRISMA-P) guideline (Shamseer et al., 2015). The protocols for the systematic reviews of EF and AF measurement tools were successfully registered separately on the PROSPERO website (see here: https://www.crd.york.ac.uk/prospero/) with registration numbers CRD42020202190 and CRD42020203968 for the EF tools systematic review and AF tools systematic review respectively.

### Eligibility Criteria

As alluded to earlier, the papers selected for critical appraisal in this systematic review were selected from the scoping review conducted earlier by the authors. The details of the eligibility criteria therefore can be found in that paper (Kusi-Mensah et al., 2021). However, in summary, we made a search for primary research papers of all study designs that focused on development or adaptation/validation of EF and AF tools used in the context of the target outcomes (executive functioning or adaptive functioning following brain pathology) among children in LAMIC countries, with no date or language restrictions. This meant that papers published from 1^st^ January 1894 (earliest date of all search engines used) to 15^th^ September 2020 (the last day of update of the search strategy) were included. The paper also had to primarily be concerned with developing, adapting or assessing the validity of the instrument of choice as one of its main stated study aims (if not the main), and not just as an incidental concern, to be eligible. For participants, studies examining children aged 5 years to 18 years (both healthy and clinical populations) living in LAMICs were included. All eligible full articles in any language were included in the search with no a priori language limits set on the search, and an attempt was made to translate non-English articles using Google Translate or volunteer native language speakers (as available). A list of potentially eligible articles that could not be obtained or translated have been provided in Appendix II. Target outcomes were defined as: 1. Papers reporting on the development (specifically concept elicitation and content validation) of a new tool assessing EF or AF; 2. Papers reporting on adaptation (content validation) of an existing HIC-derived EF or AF tool; and 3. Papers reporting psychometric properties of EF or AF tools in a LAMIC. Specifically, psychometric properties that were included as eligible for consideration were:

- Internal consistency
- Reliability (test–retest, inter-rater and intra-rater reliability)
- Validity (structural, cross-cultural, construct and criterion validity)
- Measurement Error
- Responsiveness

These measurement properties are fully defined in the ‘data items’ section below. Excluded were animal studies, studies that only used the instrument as an outcome measurement instrument (for instance in randomized controlled trials), studies in which validation was **not** of the EF or AF tool (but rather validation of another instrument for another non-EF/AF construct such as visuospatial ability, intelligence, short-term memory etc.), and all studies that did not meet the inclusion criteria.

### Information Sources

The following databases were searched with indicated dates:

1. MEDLINE (OVID interface, 1946 onwards)
2. EMBASE (OVID interface, 1974 onwards)
3. Cochrane library (current issues)
4. PsychINFO (1894 onwards)
5. Global health (1973 onwards)
6. Scopus (1970 onwards)
7. Web of Science (1900 onwards)
8. SciELO (2002 onwards): Latin America focused database providing scholarly literature in sciences, social sciences, and arts and humanities published in leading open access journals from Latin America, Portugal, Spain, and South Africa; this was an important source of non-English language studies from developing countries in Latin America, particularly from Brazil.
9. Education Resources Information Centre (ERIC, 1966 onwards)
10. British Education Index (BEI, 1996 onwards)
11. Child Development & adolescent studies (CDAS, 1927 onwards)
12. Applied Social Sciences Index and Abstracts (ASSIA, 1987 onwards): important source for multidisciplinary papers; includes social work, nursing, mental health and education journals.

GRAY LITERATURE DATA SOURCES

13. Open grey (1992 onwards): includes theses, dissertations, and teaching guides
14. PROSPERO (2011 onwards): repository of pre-registered study protocols for systematic reviews for trial protocols for similar scoping reviews through PROSPERO.
15. Cochrane library (see above)
16. EMBASE (see above)
17. ERIC (see above)
18. CDAS (see above)

In all 14 unique databases were searched initially by 20^th^ March 2020, and finally on **15**^**th**^** September 2020**. We also scanned the reference list of selected papers for other papers of possible interest which might have been missed in the literature search, particularly so for systematic and scoping review papers we found in our search.

### Search Strategy

We developed literature search strategies using text words and medical subject headings (MeSH terms) related to the following themes:

- Executive function/Frontal lobe function/Frontal lobe damage/Adaptive Function and their synonyms and variants using truncation
- Assessments/Validation/reliability/norms/reproducibility/standardization of instruments and their synonyms and variants using truncation
- Children/adolescents and their variants using truncation
- Developing countries/lower-middle-income-countries/LMIC and their synonyms and variants using truncation

The search strategy was developed by a member of the study team (KKM) who had undergone extensive training from the Medical Library Services, University of Cambridge in conducting Systematic Reviews and in using search strategies in all the above-named databases. The search strategy was also reviewed by an experienced Medical Librarian who has extensive expertise in systematic review searching. The search terms were entered sequentially first with individual terms/synonyms connected with the Boolean operator “OR” as a theme-group to broaden the inclusivity of potential hits, while theme-groups were then connected with the Boolean operator “AND” entered into the advance search function to enhance the accuracy of potential hits. The full search strategy for MEDLINE is re-produced in Appendix I (see Supplemental Material) with more details of the search strategy. This search strategy was adapted for each of the 14 databases with each of their result documented in Table 7 below (see result section).

### Study Selection

**Fig. 1:**
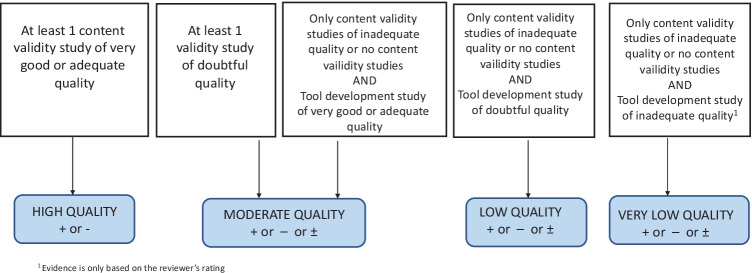
Flowchart for grading quality of evidence based on Risk of Bias (Terwee et al., 2018a, b)

This has been extensively described in the scoping review paper. Six reviewers (all authors except AB) reviewed abstracts and critically appraised all papers. A minimum of 2 reviewers independently evaluated and screened each abstract and full paper at the abstract screening and full paper screening phases and compared results at each stage. Discrepancies were resolved by discussion and mutual agreement, or where there was no agreement, by arbitration by a 3^rd^ reviewer. As a final resort, persistent disagreements were arbitrated by AB, the guarantor. Further, the pre-resolution inter-rater agreement ranged between 81.6%–88.9%, which was above the recommended minimum 80% agreement. In accordance with PRISMA recommendations, the selection process was documented in a flow diagram (see Fig. 1 below reproduced from scoping review paper) (Kusi-Mensah et al., 2021).

### Data Collection Process

At least two reviewers independently extracted data from the screened articles using purpose-made data extraction charts: for the preliminary data collection the chart was designed following the PRISMA-ScR and PRISMA-P checklists (see scoping review paper for details); while for the critical appraisal, the chart was designed using items from the risk of bias assessment tool, the **Co**nsensus-based **S**tandards for the selection of health status **M**easurement **In**struments (COSMIN) checklist items guidelines (Mokkink et al., 2018b). First a calibration exercise was done for all 6 reviewers using a sample of 100 abstracts and 10 full paper articles to ensure uniform use of the screening criteria and the charting forms. The search results were uploaded and saved into Mendeley using the ‘*groups’* function, which allowed online collaboration and discussion among the reviewers. The data were charted using custom-made Excel spreadsheets downloaded from the COSMIN website (see ‘help organising your risk of bias ratings’ here: https://www.cosmin.nl/tools/guideline-conducting-systematic-review-outcome-measures/).

### Data Items

The following data points were collected and critically appraised using the following definitions:

#### Instrument reference

this referred to the name of lead author and publication year of the paper.

#### Instrument name

this referred to the instrument name and version under consideration.

#### Outcome Variable

the outcome variables of interest were executive functioning and adaptive functioning as defined above.

#### Country Settings

the desired setting was low-and-middle-income countries (‘LAMIC’) setting which was defined according to the World Bank list of lower income country (LIC- GNI per capita less than $1025), and middle-income countries which are split into Lower-middle-Income Country (LMIC- GNI per capita between $1026 to $3995) and upper-middle-income country (UMIC- GNI per capita between $3,996 TO $12,375) list (Cochrane Library, 2012; World Bank Group, 2019).

#### Type of Study:

The critical appraisal was done according to the specific type of study done in the paper, with COSMIN criteria changing for different types of studies. ‘Validation of an assessment tool’ was defined according to specific items/criteria used for reliability and validity according to the COSMIN manual guidelines (Mokkink et al., 2014). Specific items (including their taxonomy and definitions) that were included as part of validation if they were reported upon were defined according to the COSMIN manual (see page 11, Table 1 of the manual) as follows:

- **RELIABILITY*:*** This is a domain which covers the extent to which scores for patients who have not changed on the construct in question, are the same for repeated measurement under several conditions e.g. scores not changing when one uses different sets of items from the same instrument (internal consistency); not changing over time (*test‐retest reliability*); not changing even when done by different persons on the same occasion (*inter‐ rater reliability*); or even when done by the same persons (raters or responders) on different occasions (*intra‐rater reliability*). It is a domain that measures degree to which the measurement is free from measurement error. The larger domain “RELIABILITY” comprises of the following measurement properties:
  - **Internal consistency**: The aspect of the ‘reliability’ domain which looks at the degree of the interrelatedness among the items. In other words, internal consistency is the maintenance of the same score for the same patient when different sets of items (which are related to each other) from the same instrument are used.
  - **Measurement error:** The systematic and random error of a patient’s score that is not attributed to true changes in the construct to be measured. One source of this error could be a lack of internal consistency, among other sources.
  - **Reliability:** This is a measurement property of the domain “RELIABILITY” that specifically looks at the proportion of the total variance in the measurements which is due to ‘true’ differences between patients (i.e., variance which excludes all sources of measurement error). This measurement property tests *test–retest, inter-rater* and *intra-rater reliabilities* (see above for definitions).
- **VALIDITY:** This domain refers to the degree to which an instrument measures the construct(s) it purports to measure. This domain consists of the following measurement properties:
  - **Content validity** (including **face validity**): a measurement property of validity which looks at the degree to which the content or items of an instrument is an adequate reflection of the construct to be measured within the specific cultural context in question. It usually covers the following aspects of its measurement properties: ascertaining whether each item is *relevant* (i.e. refers to concepts that are familiar and common to the experience of the target audience/likely participants), *comprehensible* (i.e. words used can be understood at the language and educational level of the target audience) and *comprehensive* (i.e. items cover all the domains or aspects of the concept as understood by the target audience).
  - **Construct Validity**: a measurement property of validity which examines the degree to which the scores of the instrument are consistent with known hypotheses (for instance with regard to internal relationships, relationships to scores of other instruments, or differences between relevant groups) based on the assumption that the instrument validly measures the construct to be measured. This will comprise of the following aspects of measurement property subsets: ***Structural Validity*** which is the degree to which the scores of an instrument are an adequate reflection of the dimensionality of the construct to be measured (i.e. if the construct is seen as a single domain, a factor analysis will not produce 2 or more factors for that same construct but just one factor reflecting that single domain); ***Cross-cultural validity***: which is the degree to which the performance of the items on a translated or culturally adapted instrument are an adequate reflection of the performance of the items of the original version of the instrument; and ***Hypothesis-testing (construct validity)***: which is the degree to which scores produced by the instrument are consistent with a known true hypothesis. Hypothesis-testing can be further divided into two sub-categories: *discriminant validation* (where the hypothesis is testing the ability of the instrument to discriminate between 2 group- say a clinical versus healthy group such as an EF instrument’s scores discriminating between a population of healthy children and children with ADHD in terms of ability to plan without distraction); *convergent validation* (where the hypothesis is testing the convergence of the instrument of interest- say an EF tool- with another instrument of related but different construct- such as an ADHD screening tool, such that there is good enough correlation between the scores produced by the two different but related tools).
  - **Criterion validity:** a measurement property of validity which looks at the degree to which the scores of an instrument are an adequate reflection of a ‘gold standard’. For outcome measurement instruments the ‘gold standard’ is usually taken as the original full version of an instrument where a shortened version is being evaluated.
- **RESPONSIVENESS:** Another broad domain which looks at the ability of an instrument to detect change over time in the construct to be measured

**Table 1:** COSMIN criteria for assessing **results** (Summary Measures) of psychometric properties (Mokkink et al., 2018b)

**Measurement Property**	**Rating**^**a**^	**Comment**
Structural Validity	+	**CTT:** CFA: CFI or TLI or NNFI or comparable measure > 0.95 OR RMSEA < 0.06 OR SRMR < 0.08^b^ **IRT/Rasch:** No violation of unidimensionality^c^: CFI or TLI or comparable measure > 0.95 OR RMSEA < 0.06 OR SRMR < 0.08 AND no violation of local independence: residual correlations among the items after controlling for the dominant factor < 0.20 OR Q3's < 0.37 AND no violation of monotonicity: adequate looking graphs OR item scalability > 0.30 AND adequate model fit: IRT: χ2 > 0.01 Rasch: infit and outfit mean squares ≥ 0.5 and ≤ 1.5 OR Z‐ standardized values > ‐2 and < 2
	?	CTT: Not all information for ‘ + ’ reported IRT/Rasch: Model fit not reported
	–	Criteria for ‘ + ’ not met
Internal Consistency	+	At least low evidence^d^ for sufficient structural validity^e^ AND Cronbach's alpha(s) ≥ 0.70 for each unidimensional scale or subscale^f^
	?	Criteria for “At least low evidence4 for sufficient structural validity^e^” not met
	–	At least low evidence^d^ for sufficient structural validity^e^ AND Cronbach’s alpha(s) < 0.70 for each unidimensional scale or subscale^f^
Reliability (test–retest, inter rater and intra- rater)	+	ICC or weighted Kappa ≥ 0.70
	?	ICC or weighted Kappa not reported
	–	ICC or weighted Kappa < 0.70
Measurement error	+	SDC or LoA < MIC^e^
	?	MIC not defined
	–	SDC or LoA > MIC^e^
Cross cultural validation/ measurement invariance	+	No important differences found between group factors (such as age, gender, language) in multiple group factor analysis OR no important DIF for group factors (McFadden's R^2^ < 0.02)
	?	No multiple group factor analysis OR DIF analysis performed
	–	Important differences between group factors OR DIF was found
Hypotheses testing: construct val	+	The result is in accordance with the hypothesis^g^
	?	No hypothesis defined (by the review team)
	–	The result is not in accordance with the hypothesis^g^
Criterion validity	+	Correlation with gold standard ≥ 0.70 OR AUC ≥ 0.70
	?	Not all information for ‘ + ’ reported
	–	Correlation with gold standard < 0.70 OR AUC < 0.70
Responsiveness	+	The result is in accordance with the hypothesis^g^ OR AUC ≥ 0.70
	?	No hypothesis defined (by the review team)
	–	The result is not in accordance with the hypothesis^g^ OR AUC ≤ 0.70

*AUC* area under the curve, *CFA* confirmatory factor analysis, *CFI* comparative fit index, *CTT* classical test theory, *DIF* differential item functioning, *ICC* intraclass correlation coefficient, *IRT* item response theory, *LoA* limits of agreement, *MIC* minimal important change, *RMSEA* Root Mean Square Error of Approximation, *SEM* Standard Error of Measurement, *SDC* smallest detectable change, *SRMR* Standardized Root Mean Residuals, *TLI* Tucker‐Lewis index

^a^“ + ” = sufficient,”– “ = insufficient, “?” = indeterminate

^b^To rate the quality of the summary score, the factor structures should be equal across studies

^c^unidimensionality refers to a factor analysis per subscale, while structural validity refers to a factor analysis of a (multidimensional) patient‐reported outcome measure

^d^As defined by grading the evidence according to the GRADE approach

^e^This evidence may come from different studies

^f^The criteria ‘Cronbach alpha < 0.95’ was deleted, as this is relevant in the development phase of a PROM and not when evaluating an existing PROM

^g^The results of all studies should be taken together, and it should then be decided if 75% of the results are in accordance with the hypotheses

The above definitions of measurement properties also largely conformed with the definitions given in The Standards for Educational and Psychological Tests manual American Educational Research Association, American Psychological Association, & National Council on Measurement in Education, 2018). Any paper that reported information on any of the above was considered as eligible for having included an eligible outcome measure. An important judgement decision that was made in respect of classification of what constituted “**a study**” was that any individual validation conducted in any given research project/paper was regarded as a “study” in accordance with COSMIN manual recommendations (Prinsen et al., 2018). So, for example, a given paper might report the conduct of say- construct validation (hypothesis testing), cross cultural validation and structural validation of one instrument all within the same paper. This was thus reported as three studies reported within one paper.

**Mode of administration:** This was defined as the way in which the instrument was administered, i.e. whether the instrument was a performance-based task, or an informant-based tool (i.e., self-reported or parent/proxy-based questionnaire etc.). For performance-based tasks, the number of trials of a task was considered as the “number of items” when it came to evaluating aspects like adequacy of sample size in Structural validity or Cross-cultural validity studies.

**Sub-domains:** the number of sub-scales or sub-domains or items of interest of the tool in question reported on.

**Sample size:** number of participants used.

**Demographics:** mean age and gender percentages of sample.

**Local Settings:** whether study was predominantly set in rural or urban settings (or both).

**Condition:** whether study was conducted among health sample or clinical sample, and if so, what clinical condition.

**Language of population:** What local language-group was the study conducted among.

### Risk of Bias in Individual Studies

Each study was evaluated for both the quality of its methodology (Risk of bias assessment) and the quality of its results following the COSMIN guidelines (Mokkink et al., 2018a). This COSMIN guidelines came in 2 separate manuals: 1. *The COSMIN methodology for assessing the content validity of Patient‐Reported Outcome Measures (PROMs)* (C. B. ) which focuses on assessing risk of bias in content validity studies, and 2. *the COSMIN methodology for systematic reviews of Patient‐Reported Outcome Measures (PROMs)* (Prinsen et al., 2018) which focuses on risk of bias for all other types of validity studies. This section describes the risk of bias rating for the **methodology** used while the next section describes that of *results*. The risk of bias evaluation was done at both the study level and the instrument level. A critical appraisal of each study was done independently by at least 2 reviewers and compared, and consensus reached. The methodology was rated using 4 codes: ‘very good’, ‘adequate’, ‘doubtful’ and ‘inadequate’. So, for example, in evaluating a Structural Validity study for an instrument developed using classical test theory (CTT), the first item to be evaluated in the methodology would be whether Confirmatory Factor Analysis (CFA) was performed (scored as: ‘very good’), as opposed to Exploratory Factor Analysis (EFA score: “adequate”), or no CFA or EFA was performed at all (score: “inadequate”). For giving the overall rating of each study, the ‘worst score count’ system was used as per the COSMIN guideline, for the simple reason that “poor methodological aspects of a study cannot be compensated by good aspects” (Mokkink et al., 2018a; Caroline B ). For a detailed explanation of the individual criteria for each respective rating in all the scoring domains refer to pages 47 – 63 of the ‘COSMIN Manual for Systematic Reviews of PROMs’ (Mokkink et al., 2018a) which is downloadable for free here: https://www.cosmin.nl/tools/guideline-conducting-systematic-review-outcome-measures/.

Further, any qualitative studies for content validation found for new tool development or adaptation studies were critically appraised using the separate COSMIN guidelines for evaluation of content validation studies (Terwee et al., 2018a, b) (which is different from the COSMIN manual whose methodology was “developed in 2016 in a Delphi study among 158 experts from 21 countries”. This is also downloadable for free at the above URL of the COSMIN website. Here also the methodology of the content validation/instrument development study was appraised for risk of bias (also rated as: ‘very good’, ‘adequate’, ‘doubtful’ and ‘inadequate’- see COSMIN box 1 and box 2 in excel spreadsheet in supplemental material). The extracted and critically appraised data were then summarised per each identified instrument, and an overall rating given to the quality of evidence according to the COSMIN guideline, using the GRADE (Grading of Recommendations Assessment, Development, and Evaluation) approach (Schünemann et al., 2008), as described below (see Table 2 below).

**Table 2:** COSMIN criteria and rating system for evaluating the content validity of Instruments (Terwee et al., 2018a, b)

**Name of the Instrument or subscale:** ………………………….	**Instrument development study**	**Content validity study 1**	**Content validity study 2**^**b**^	Rating of reviewers^*^	**OVERALL RATINGS PER PROM**^**c**^** (see step 3b in COSMIN manual)**	**QUALITY OF EVIDENCE (see step 3c in COSMIN manual)**
Criteria (see Table… in Appendix for guidelines)	+ /–/ ± /?^a^	+ /–/ ± /?^a^	+ /–/ ± /?^a^	+ /–/ ± /?^a^	+ /–/ ± /?^a^	High,moderate, low, very low
**Relevance**						
1. Are the included items relevant for the construct of interest?^d^						
2. Are the included items relevant for the target population of interest?^d^						
3. Are the included items relevant for the context of use of interest?^d^						
4. Are the response options appropriate?						
5. Is the recall period appropriate?						
**RELEVANCE RATING (see COSMIN page 58, Table **3**)**						
**Comprehensiveness**						
6. Are all key concepts included?						
**COMPREHENSIVENESS RATING (see COSMIN page 58)**						
**Comprehensibility**						
7. Are the Instrument instructions understood by the population of interest as intended?						
8. Are the Instrument items and response options understood by the population of interest as intended?						
9. Are the Instrument items appropriately worded?						
10. Do the response options match the question?						
**COMPREHENSIBILITY RATING (see COSMIN page 58)**						
**CONTENT VALIDITY (see COSMIN page 59, Table **4**)**						

^*^Rating of Reviewers excluded from this review for reasons stated above

^a^Ratings for the 10 criteria can only be + / – /?. The RELEVANCE, COMPREHENSIVENESS, COMPREHESIBILITY, AND CONTENT VALIDITY ratings can be + / – / ± /?

^b^Add more columns if more content validity studies available. ^3^If ratings are inconsistent between studies, consider using separate tables for subgroups of studies with consistent results

^c^These criteria refer to the construct, population, and context of use of interest in the systematic review

**Table 3:** Modified GRADE approach for grading the quality of evidence (Mokkink et al., 2018)

**QUALITY OF EVIDENCE**	**LOWER IF**
High	Risk of bias:
	-1 Serious
Moderate	–2 Very serious
	–3 Extremely serious
Low	
	Inconsistency:
Very Low	–1 Serious
	–2 Very serious
	Imprecision: –1 total n = 50–100 –2 total n < 50 Indirectness: –1 Serious –2 Very serious

**Table 4:** Instructions on Downgrading for Risk of Bias (Mokkink et al., 2018a)

**Risk of bias**	**Downgrading for Risk of Bias**
No	There are multiple studies of at least adequate quality, or there is one study of very good quality available
Serious	There are multiple studies of doubtful quality available, or there is only one study of adequate quality
Very serious	There are multiple studies of inadequate quality, or there is only one study of doubtful quality available
Extremely serious	There is only one study of inadequate quality available

### Summary Measures

This section describes evaluation of **results** reported by the papers. The principal summary measures to be evaluated across studies differs according to study type. Table 1 below (reproduced from COSMIN manual for Systematic Reviews) summarises the various summary measures and criteria for all study types except content validity (reported later). After rating the methodology, the results reported were then rated as: ‘ + ’ for sufficient, ‘– ‘for insufficient and ‘?’ for indeterminate. For the construct validation, the acceptable measure of effect of the instrument of interest when compared with another similar instrument was pre-defined by the study team as an expected correlation between the two of at least 0.5 as recommended by the COSMIN guideline (Mokkink et al., 2018a). For comparison of healthy versus clinical populations on EFs or AFs, the pre-defined minimum acceptable difference was a statistically significant difference (Mokkink et al., 2018a) with an odds-ratio of at least 1.5. The results for individual assessment tools from different papers were then qualitatively pooled together and given an overall rating also according to ‘sufficient’, ‘insufficient’ and ‘indeterminate’.

For the content validation, results were rated as: ‘ + ’ for sufficient, “– “ for insufficient, ‘?’ for indeterminate and “ ± “ for inconsistent. The general rule for giving a sufficient rating per criterion was as follows:

- +  ≥ 85% of the items of the instrument (or subscale) fulfill the criterion.
– < 85% of the items of the instrument (or subscale) does fulfill the criteria.
- ?No(t enough) information available or quality of (part of a) the study inadequate.
- ± Inconsistent results.

The ten (10) individual criteria for synthesizing results of Content Validity are specified in Table 2 below. The results of each criterion are rated taking into consideration the risk of bias assessment (i.e., quality of methodology- which would have been rated in COSMIN box 1 and box 2) and rated accordingly. The results rating was recorded in Table 2 below.

In using the COSMIN content validation manual (Terwee et al., 2018a, b, we made the following modifications for the following reasons. Firstly, although the COSMIN content validation manual allowed for three types of ratings to be assessed- 1. rating of original development study (if available or relevant) 2. rating of all adaptation or content validation studies, and 3. independent rating of all items by reviewers- following which a summary score would be given for the content validity of the instrument (see page 52 of COSMIN content validity manual), we chose to drop the third type of rating (the reviewers independent rating) and rather just stick with the first two- development study rating and adaptation/content validity studies rating. We followed this course of action for two reasons: firstly obtaining the actual instrument under review (rather than just the published validation study on that instrument) was not always going to be practical or even possible sometimes since several of them were copyrighted material under commercial licence for which we would have to pay a fee to obtain the instrument; the decision was therefore made that if we were not going to be able to obtain the full instrument for ALL eligible papers, then it would be unfair to evaluate some instruments on those three levels, and compare these with others that were evaluated on just 2 or even 1 level simply because some instruments were freely available and others were not.

Secondly, given our varying levels of expertise/experience and given the wide variety of countries from which we were going to evaluate papers, we also wanted to further minimise the amount of subjectivity that would go into us personally reviewing each item on candidate instruments as to the appropriateness of items for each country across such a diverse array of country-specific sociocultural realities and languages that we were not personally familiar with; and then going on to factor in these subjective impressions into the overall rating of the instrument. We therefore thought it would be better to leave the review of the appropriateness of individual items for individual country contexts (if done at all) to the local experts who might have been involved with the individual projects. We thus thought it best to restrict ourselves to only the review of the published development and adaptation/content validation papers using the specified standard criteria published in the two COSMIN manuals across board. This we felt would be more objective and give all tools an even playing field.

A practical effect of this approach in determining the OVERALL RATING of results of an instrument (summing up ALL available studies) was as follows: the COSMIN manual states that since there is supposed to be a Reviewers’ independent rating of each item (apart from the rating of the published development study and rating of the adaptation studies), where the results of the development and adaptation studies are at variance, the Reviewers’ own independent rating should be used, hence it should not be possible to give an ‘indeterminate’ OVERALL RATING for any instrument (see page 60 of COSMIN content validity manual). However, contrary to this, we *did* make it possible to give an 'indeterminate' overall results rating ('?') where the rating for the development study and the adaptation study were at variance since we did not do any Reviewers' rating to fall back on for an overall rating.

Another modification that was made in the application of the COSMIN criteria was in the risk of bias (RoB) evaluation for performance-based tasks. In evaluating the content validity of performance-based tasks, we did not deem it appropriate to assess "comprehensiveness" of the task (how well does the task cover all aspects of the construct at hand) among the caregivers/patients, because being lay people it seemed a bit unfair and unreasonable to ask them to evaluate how comprehensive such highly specialized tasks were to the construct of interest. Therefore, we only evaluated “comprehensiveness" among subject matter experts. However, for such domains as “relevance”, we went ahead and evaluated these performance-based tasks for relevance among caregivers/patients as per COSMIN guidelines because we felt that asking about the relevance of a task to the experience of a patient/caregiver was perfectly legitimate in such circumstances. As noted by Semrud-Clikeman and colleagues, when a task is unfamiliar to a child in a LAMIC but familiar to children in Western cultures, administering such a task to the LAMIC child may measure the ability of LAMIC children to adapt to new situations rather than their ability to complete the actual task, rendering the scores invalid for the domain being measured (Semrud-Clikeman et al., 2017), hence the need to assess even performance-based tasks for relevance.

A final modification made in applying COSMIN was in evaluating the RECALL PERIOD (see item 5 in Table 2 below). Because behaviours being described by the two constructs we evaluated (Executive and Adaptive functioning) were not "symptoms" per se, items concerning RECALL PERIOD were deemed as 'NA- not applicable'. This was because given that these behaviours were not symptoms with a "time of onset", but rather normal behaviours expected at various age-appropriate milestones, it did not seem suitable to evaluate whether authors concerned themselves with recall periods of how far back the item was to be evaluated and would thus have been unfair to have penalized them for not doing so (as per COSMIN guidelines) given the context. Further, for performance-based tasks only, evaluation of appropriateness of RESPONSE OPTIONS (see item 4 in Table 2 below) such as ‘often’, ‘sometimes’, ‘never’ etc. as seen in questionnaires was also deemed ‘not applicable’. This question while making perfect sense in the context of an informant-based instrument (which has various response options such as ‘sometimes’, ‘never’, ‘often’ etc.), would not make sense in the context of a performance-based tasks such as Go/No-go or Wisconsin Card Sorting Test where the response is an action.

### Synthesis of Results

This section describes how methodology and results ratings of individual studies were synthesized and summarized across several papers for each instrument. After rating the methodology and results of individual studies and qualitatively pooling these together to give an overall rating per instrument, the overall **quality of the evidence** for the reported results of the instrument in question (taking into consideration all published papers for that instrument) was then graded following the GRADE (Grading of Recommendations Assessment, Development, and Evaluation) approach (Schünemann et al., 2019). In other words, after giving the overall rating of the results of a study type per instrument, this result was then accompanied by a grading of the quality of evidence using the GRADE approach.

The instrument in question is usually assumed to be of high quality of evidence from the start (see Table 3 below reproduced from page 34 of COSMIN manual (Mokkink et al., 2018a)**)**, and progressively downgraded to lower levels, taking into consideration the best rating for Risk of Bias, inconsistencies of results from different studies (i.e. between study variability/heterogeneity), imprecision of results (i.e. down-grading for low pooled sample sizes) and indirectness (i.e. downgrading for studies that were (partly) performed in another population or another context of use than the population or context of use of interest in the systematic review, for example, evidence from a mixed sample with adults rather than just children and adolescents).

The final quality of evidence was then graded as: ‘high’, ‘moderate’, ‘low’ or ‘very low’ depending on which level the instrument was finally left at following successive downgrades. This grading serves to indicate how confident one can be that the overall rating is trustworthy (Terwee et al., 2018a, b). So, for example, in this scheme, 2 different tools- Instrument A and Instrument B- might both have a rating of “ + ” (sufficient) in their structural validity, but Instrument A might have a grading of “high” for the quality of evidence supporting its rating of “ + ”, while Instrument B might have a grading of “very low” for the quality of evidence supporting its own “ + ” rating.

To go into more detail, Risk of Bias (RoB) was given an overall grade of “No risk”, “serious”, “very serious” or “extremely serious” risk depending on the situation specified against each grade in Table 4 below (reproduced from page 34 of COSMIN manual (Mokkink et al., 2018)). So, for example, an instrument that had multiple studies of ‘inadequate’ quality, or only one study of ‘doubtful’ quality in its methodology rating would be rated as having ‘very serious’ RoB (see Table 3) and would be downgraded by –2 steps from say “High” quality of evidence to “Low” quality of evidence according to GRADE criteria (see Table 4).

A similar principle of progressive downgrading was followed for Inconsistency of Results. The pooled or summarized results were rated based on a majority of individual (study) results as ‘sufficient’ or ‘insufficient’ (i.e., if most consistent results were ‘sufficient’ overall rating would be sufficient, if majority of consistent results were ‘insufficient’, overall rating for consistency would be ‘insufficient’) and then downgraded accordingly using the GRADE approach in Table 3. For the purposes of determining the degree to which the downgrade was to be made for inconsistency, COSMIN recommended that the review team come to consensus about that decision. Consequently, the team decided that a downgrade of –1 (‘serious’) would be made for inconsistency if a super majority of results (above 75%) were consistent, in which case the results would be rated as ‘sufficient’ but quality of evidence would be downgraded by only –1 (say from ‘high’ to ‘moderate’) for inconsistency. However, if less than 75% but more than 50% of studies found agreement (e.g., 60% of studies gave ‘sufficient’ results and 40% gave ‘insufficient’ results for any given parameter- say, Test–retest reliability) then the overall rating would be given as “sufficient” but quality of evidence downgraded by –2 levels (‘very serious’) say from “High” to “Low” quality. If less than 50% of studies gave a consistent result (e.g., 40% of studies gave ‘sufficient’ and 60% gave ‘insufficient’ results) then the overall rating would be given as ‘insufficient’ but quality of evidence downgraded by –2 levels.

Having given this explanation, it must be stated that a slight departure was made in applying this rule when it came to Construct Validation, though this alternative approach was still in accordance with COSMIN recommendations (see page 34 of COSMIN manual). For Construct validation, where the two hypotheses being tested were of a fundamentally different nature (e.g., discriminant validation, as opposed to say a convergent validation), the strategy adopted was to summarize the results by sub-groups (i.e. summarize according to each individual hypothesis) rather than pooling all the results together. So, in this case, the results of all convergent validity studies will be pooled separately from the results of all discriminant validation studies treating both as “sub-groups” of construct validation, rather than pooling together all “construct validity” studies and using the 75% majority rule.

For Imprecision, the total sample size of all the included studies (on the validation study in question) were simply pooled together and the quality of evidence simply downgraded with one level when the total sample size of the pooled or summarized studies was below 100, and with two levels when the total sample size was below 50, as per the COSMIN manual (see page 35) (Mokkink et al., 2018). For Indirectness, this was typically not a major concern in this study since part of the screening criteria was that the study be performed primarily among children (aged 5 – 18 years). However, in the few instances where there were mixed study populations involving say young adults (19 – 25 years), only a single level downgrade (‘serious’) of quality of evidence was made, as recommended by the COSMIN manual.

This downgrading was done successively from “high” downwards following evaluation of ‘risk of bias’, ‘inconsistency of results’, ‘imprecision’ and ‘indirectness’ as explained above. The results of the quality of evidence for all instruments were then summarized and presented below in the results section. The interpretation of what each of the ratings (individual studies) and the grading (overall quality of evidence for individual instruments) is shown in Table 5 below.

**Table 5:** COSMIN Ratings/grade and their Interpretations (Mokkink et al., 2018b)

**RATINGS**	**DEFINITION/INTERPRETATION**
**Tool Development/Adaptation**^**a**^	
+ or ‘sufficient’	≥ 85% of the items of the instrument (or subscale) fulfil the criterion
– or ‘insufficient’	< 85% of the items of the instrument (or subscale) does fulfil the criteria
? or ‘indeterminate’	Not enough information available or quality of (part of) the study inadequate
± or ‘inconsistent’	Not used in rating individual items/criteria, but only used in rating the RELEVANCE, COMPREHENSIVENESS, COMPREHESIBILITY, and overall CONTENT VALIDITY of a study where results for each section are mixed
**GRADE quality of evidence**	
High	We are very confident that the true measurement property lies close to that of the estimate* of the measurement property
Moderate	We are moderately confident in the measurement property estimate: the true measurement property is likely to be close to the estimate of the measurement property, but there is a possibility that it is substantially different
Low	Our confidence in the measurement property estimate is limited: the true measurement property may be substantially different from the estimate of the measurement property
Very Low	We have very little confidence in the measurement property estimate: the true measurement property is likely to be substantially different from the estimate of the measurement property

These definitions were adapted from the GRADE approach (Schünemann et al., 2019)

^*^Estimate of the measurement property refers to the pooled or summarized result of the measurement property of an instrument

^a^refers to assessment of ‘relevance’, ‘comprehensibility’ and ‘comprehensiveness’

For the synthesis of results of the content validation, following the separate COSMIN guidelines for evaluation of content validation studies (Terwee et al., 2018a, b) a similar but slightly different approach was used for results synthesis. First, as stated above with the other types of validations, each criterion for results of content validity was given a rating (± /?) that took into consideration the methodology of content validation rating (see COSMIN box 1 and box 2 in Excel spreadsheet in supplemental material), which rating was recorded in the corresponding item in Table 2. After rating each criterion, a summary rating is then given to the RELEVANCE box (criteria 1 – 5), COMPREHENSIVENESS box (criteria 6), and COMPREHENSIBILITY box (criteria 7 – 8) as either ± /?/ ± (see COSMIN content validity manual, page 58, Table 3 for rules for summarising rating per box).

Then, a CONTENT VALIDITY rating is given **for the study** according to COSMIN guidelines (see COSMIN content validity manual, page 59, Table 4 for rules for summarising content validity rating per study) also as ± /? / ± . Finally, the content validity ratings from all available studies are summarised **for the instrument** and an overall quality of evidence is given according to GRADE approach as high’, ‘moderate’, ‘low’ and ‘very low’, all following the specified COSMIN guidelines (see steps 3b and 3c of COSMIN Content Validity Manual on page 60–62).

This overall rating is done factoring in both the score of the content validity of the eligible adaptation study/studies under consideration, as well as the score of the content validity of the original instrument development study of that instrument following a critical appraisal as per usual COSMIN guidelines whether or not this original development study was captured by the search strategy. This would mean attempting to obtain the original instrument development study and appraising it, even where it would not otherwise fall into the eligibility criteria (for example, when appraising instruments developed in HICs that were being adapted for use in a LAMIC- the original development study (in the HIC) will have to be critically appraised, and the score factored into the **overall content validity rating of the instrument)**. Where the original development study could not be obtained (for whatever reasons) to be critically appraised, an estimation of their probable score based on publicly available information and an assumption of the best case scenario of their likelihood of fulfilling (or failing to fulfil) the COSMIN criteria was made, and this was then used in estimating their overall content validation score while factoring in the score of the adaptation studies that were eligible as just described, with cogent reasons provided for their estimated score. Wherever this type of estimation was done, this has been clearly indicated in the results and in the supplemental material for the sake of transparency.

The criteria for rating quality of evidence are reproduced in Table 6 and Fig. 1 below. Because of the decision to exclude ‘Reviewer rating’ level, the COSMIN guideline for summarising and giving an overall content validity rating per instrument was modified to allow for an ‘indeterminate’ rating (?). As recommended by COSMIN, where multiple content validity studies were reported for any single instrument, ‘indeterminate’ studies were ignored, and only ‘sufficient’ or ‘insufficient’ results were considered and summarised. However where only a single study of ‘indeterminate’ result was found for content validity, rather than ignore the “?” rating, we chose to report this (?) as the final summary rating. The quality of evidence was then determined as ‘high’, ‘moderate’, ‘low’ and ‘very low’ according to the modified GRADE approach as outlined in COSMIN guidelines.

**Table 6:** Modified GRADE approach for grading the quality of evidence for Content Validity (Terwee et al., 2018a, b)

**STUDY DESIGN**	**QUALITY OF EVIDENCE**	**LOWER IF**
At least 1 content validity study	High	Risk of bias: –1 Serious –2 Very serious –3 Extremely serious Inconsistency: –1 Serious –2 Very serious Indirectness: –1 Serious –2 Very serious
No content validity studies	Moderate	
	Low	
	Very Low	

Downgrading the GRADE quality of evidence rating for risk of bias was done as follows. Downgrade for ‘serious RoB’ (–1 level, e.g., from high to moderate) was made if the adaptation/content validity study was of ‘doubtful’ methodological quality. If there were no content validity studies (or only of inadequate quality) and the instrument development study was of doubtful quality, downgrade for ‘very serious RoB’ (–2 levels) was made. If there was no adaptation/content validity study (or if only one of ‘inadequate’ quality) and the instrument development study was also of ‘inadequate’ quality, RoB downgrade was –3 levels (extremely serious) to ‘very low’ quality of evidence. Figure 1 (reproduced from COSMIN content validity manual) summarizes the flowchart for downgrading for RoB. For inconsistency, downgrade to ‘serious’ (–1 level) was made if the results rating for the instrument development study and the adaptation/content validation study were inconsistent.

### Risk of Bias Across Studies

Meta-biases like publication bias were reduced by searching the ‘grey literature’ as indicated above. However, it was difficult to guard against selective reporting within studies as this was a largely qualitative systematic review which therefore precluded requesting for original datasets from authors to conduct a “meta-analysis” of results.

## Results

### Study Selection

Table 7 (reproduced from the scoping review) summarises the results of the initial search in each individual data source, along with the dates of coverage of the search in each database.

**Table 7:** Database Results and Dates

**Database**	**Date of search coverage**	**Initial search** by 20/03/20	**Initial de- duplication**	**Updated search** by 15/09/20	**Updated de- duplication**
MEDLINE via OVID	1946 – 05/09/20	803	662	939	760
EMBASE^a^	1974 – 05/09/20	27	26	29	27
Cochrane Library^a^	^*^11/09/20	67	49	68	48
PsychINFO	1894 – 11/09/20	1314	985	1490	812
Global Health	1973 – 11/09/20	38	14	38	13
Scopus	1970 – 11/09/20	1093	1080	1134	910
Web of Science	1900 – 11/09/20	1730	1254	1820	1184
SciELO	2002 – 15/09/20	29	28	29	26
ERIC^a^	1966 – 15/09/20	19	2	19	2
BEI	1996 – 15/09/20	2	2	3	1
CDAS^a^	1927 – 15/09/20	13	3	13	2
ASSIA	1987 – 15/09/20	84	81	87	48
Open Gray^a^	1992 – 15/09/20	4	4	4	4
PROSPERO^a^	2011 – 15/09/20	1	0	2	0
Other (reference lists etc.)	–	–	–	0	0
**TOTAL ABSTRACTS**				**5675**	**3837**

*ERIC* Education Resources Information Centre, *ASSIA* Applied Social Sciences Index and Abstracts, *CDAS* Child Development & Adolescent studies, *BEI* British Education Index

^*^Although first established in 1996, composite nature of Cochrane library means it does not have a “start date” as other biomedical databases do

^a^Includes theses, dissertations, teaching guides and other such non- peer-reviewed “grey” literature

**Fig. 2:**
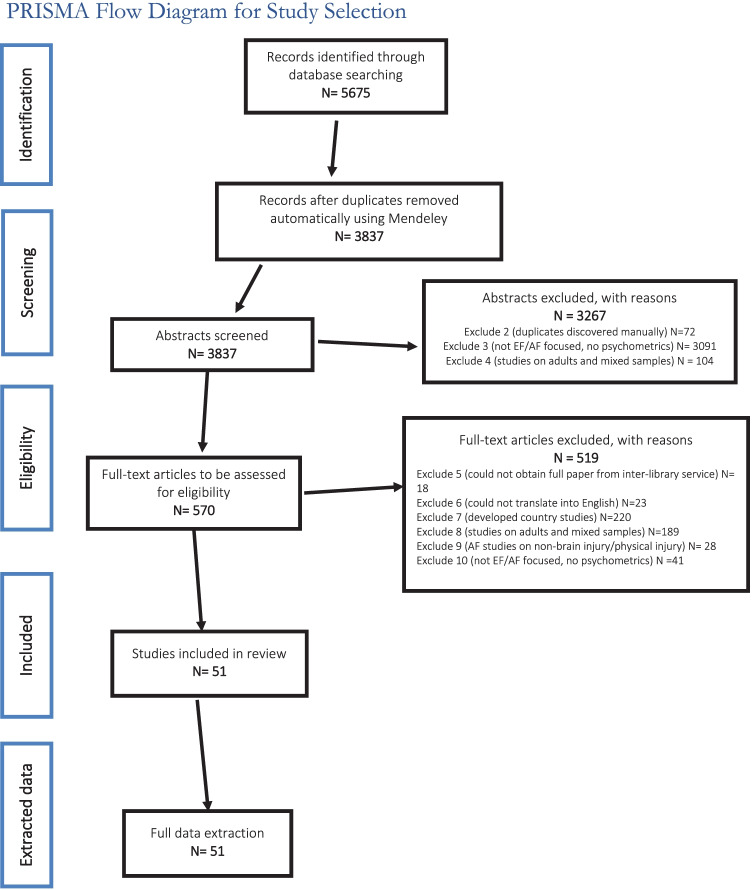
PRISMA flow diagram of study selection

Following de-duplication, manual screening of full papers for eligibility, 51 full papers were found to be eligible for full data extraction and critical appraisal. Web of Science showed a surprisingly higher number of results than PsychINFO possibly because of the search strategy that was used: because the search terms, ab initio, did not preclude studies that only used these assessment tools in psychometrically unrelated papers without necessarily reporting psychometric properties (these were later excluded on manual inspection of abstracts and full review papers). Therefore, Web of Science, which is a database dedicated to multidisciplinary research (and hence also likely to have a lot more abstracts than the more specialised PsychINFO), was likely to include a significant proportion of abstracts of such “use studies” which were later excluded as ineligible upon manual screening by the reviewers. The screening process and reasons for exclusion are summarised in the PRISMA flow diagram below in Fig. 2.

## PRISMA Flow Diagram for Study Selection

### Study Characteristics

Details of the study characteristics of each paper are reported elsewhere in the scoping review paper by the authors (Kusi-Mensah et al., 2021), and provided in the Appendix of this paper. However, some key highlights are pertinent here. A total of 163 studies (as defined under *Data Items* above) were reported in 51 papers. The most frequently conducted types of study were Structural Validity and Construct Validity/Hypothesis testing studies at 38 studies each (23.3% each of total individual studies), followed by Internal consistency studies at 27 (16.6%). In terms of geographical regions, the Americas (Central and South America) (30.4% of papers), Sub-Saharan Africa (21.4% of papers) and the Middle East (17.9% of papers) were the top 3 performing regions in terms of number of papers published from LAMICs. Most studies conducted involved urban populations at (76.7%), and exclusively healthy populations (60.8%) as opposed to clinical populations (with health controls).

### Results: Risk of Bias Within and Across Studies and Results of Individual Studies

Table 8 shows a summary of the results (synthesis of results for all studies for an instrument pertaining to each measurement property) of the critical appraisal for all the types of validity except content validity (see Table 9 for content validity results). The quality of evidence was summarised using the GRADE approach (Schünemann et al., 2019) shown by the colour coding in the table. For the details of the rating of individual studies, see the following boxes in the Supplemental Material: for Risk of Bias of various measurement properties of validations in individual studies- COSMIN boxes 3–10, for results of individual studies- COSMIN Supplemental Table 2; for details of the reasons behind each rating, see comments on ‘Summary Psychometric Ratings’ in Supplemental Material.

**Table 8:**
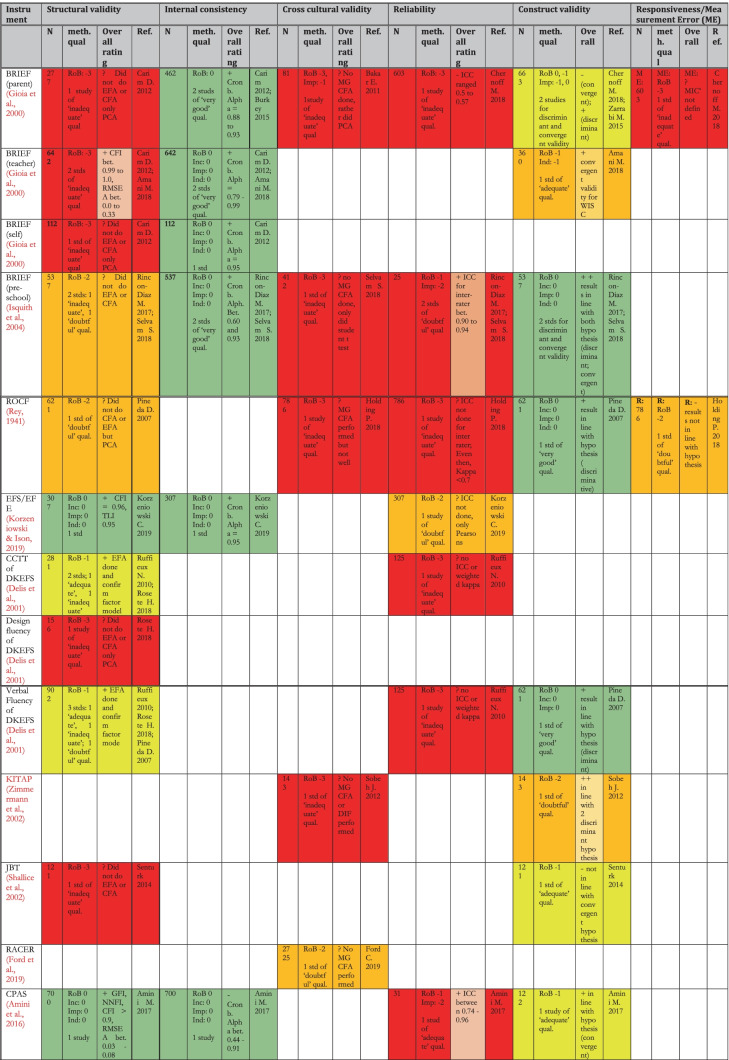

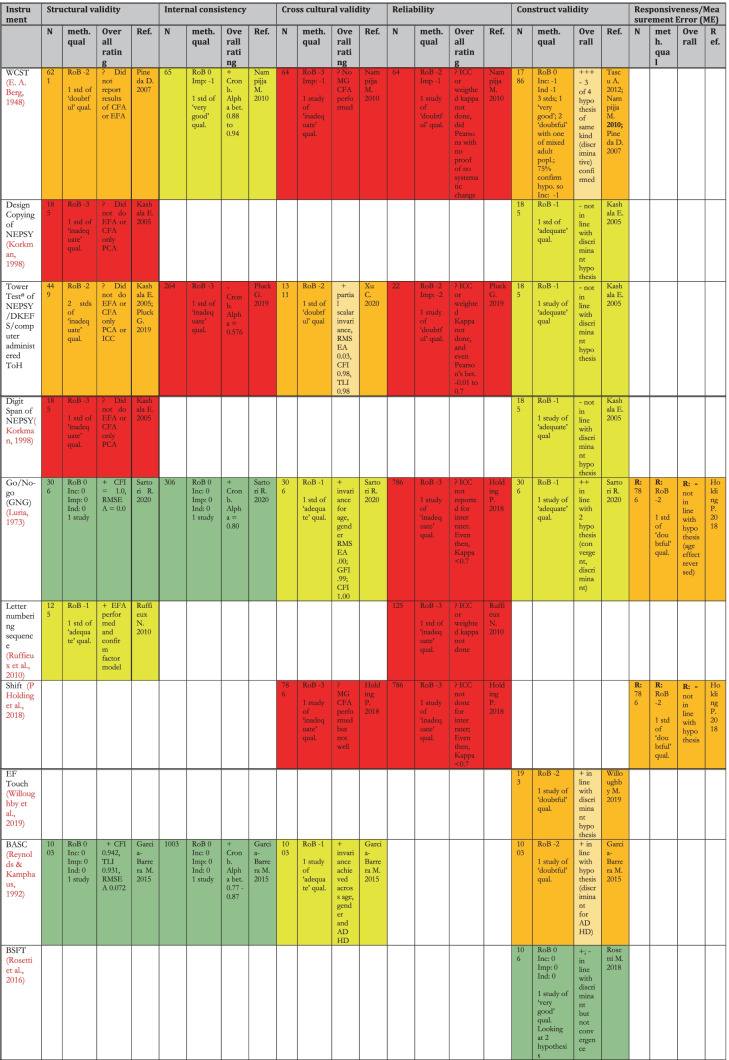

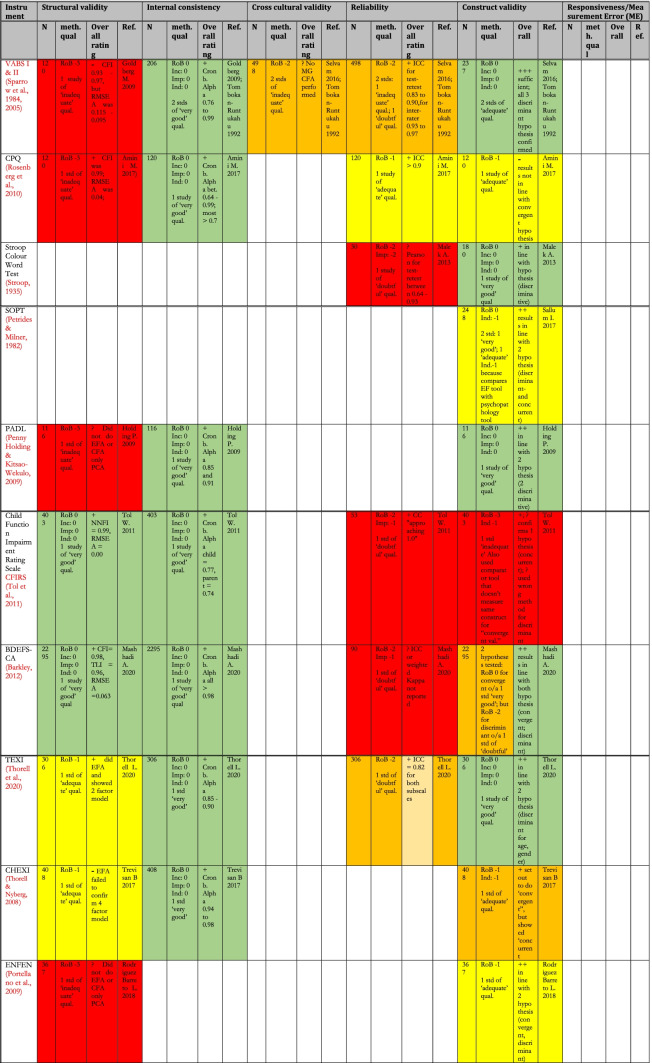

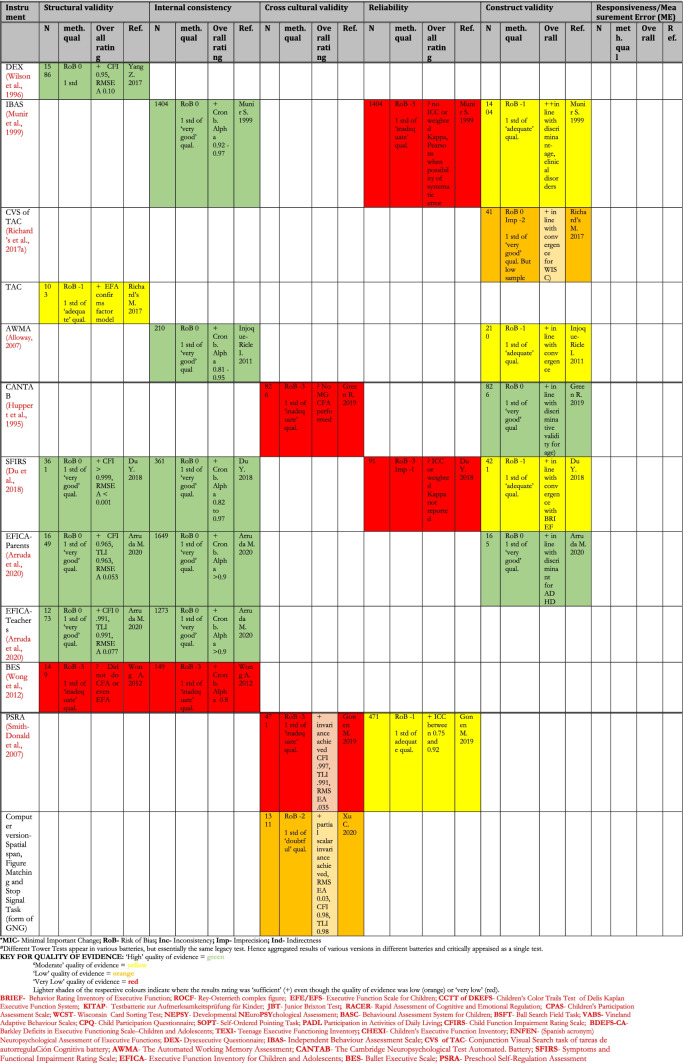
Summary of Results (Synthesis of Results) for Critical Appraisal of Psychometrics of Executive Functions and Adaptive Function tools Validated for use Among Children in LAMICs

**Table 9:**
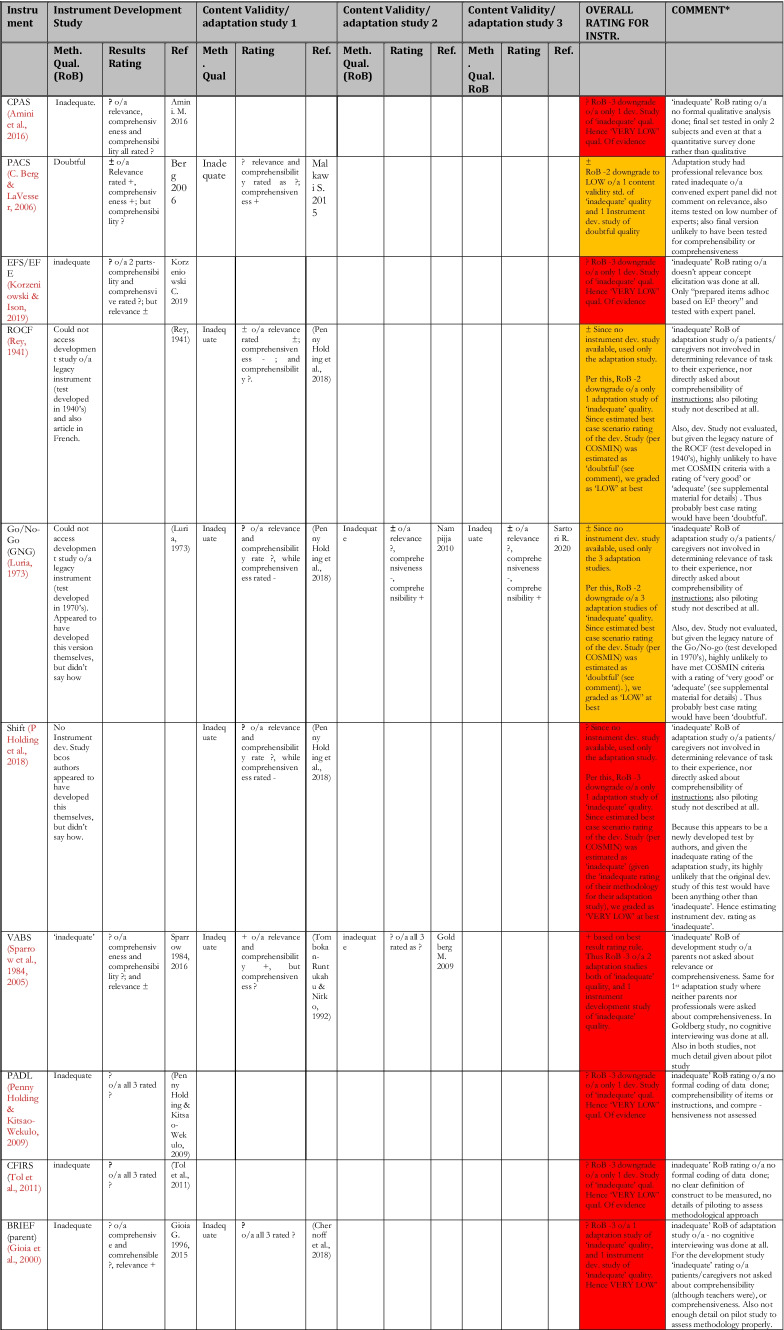

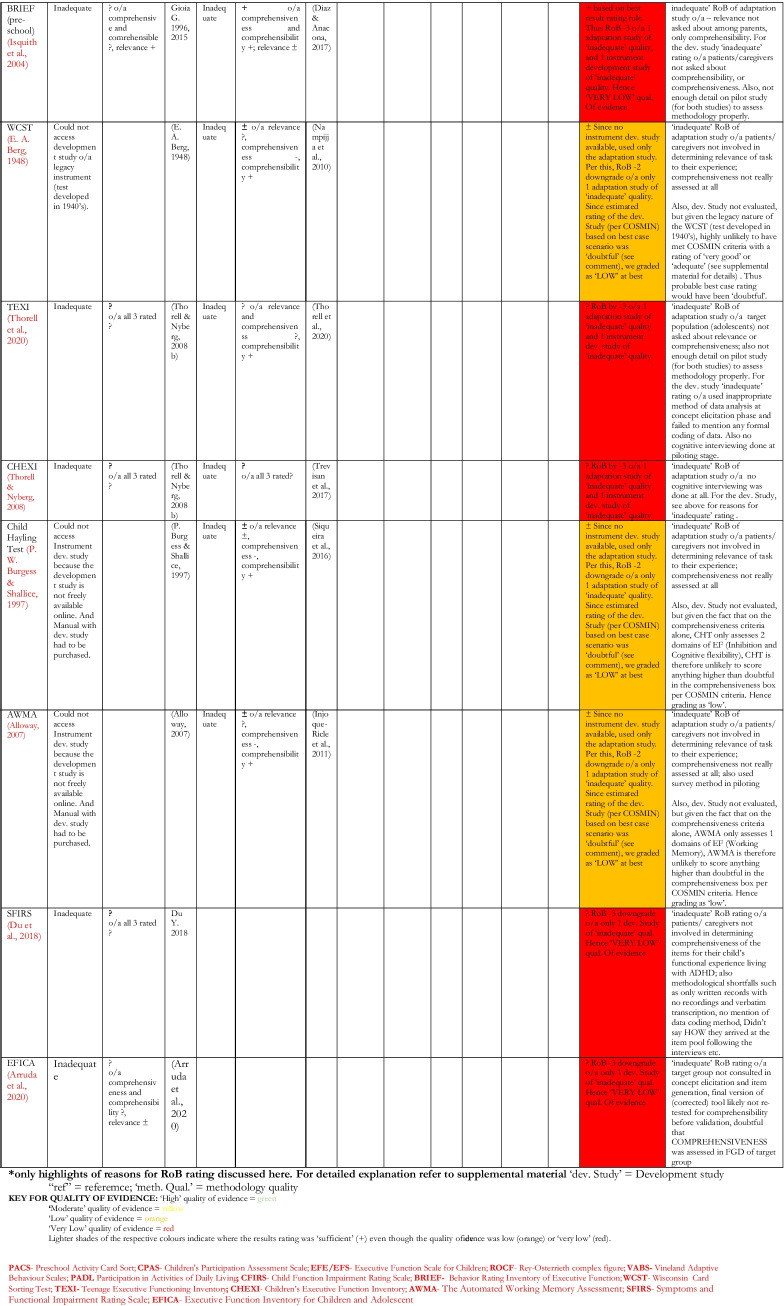
Summary of Results of Critical Appraisal of Content Validation/Adaptation of EF and AF tools Validated for use Among Children in LAMICs

**Fig. 3:**
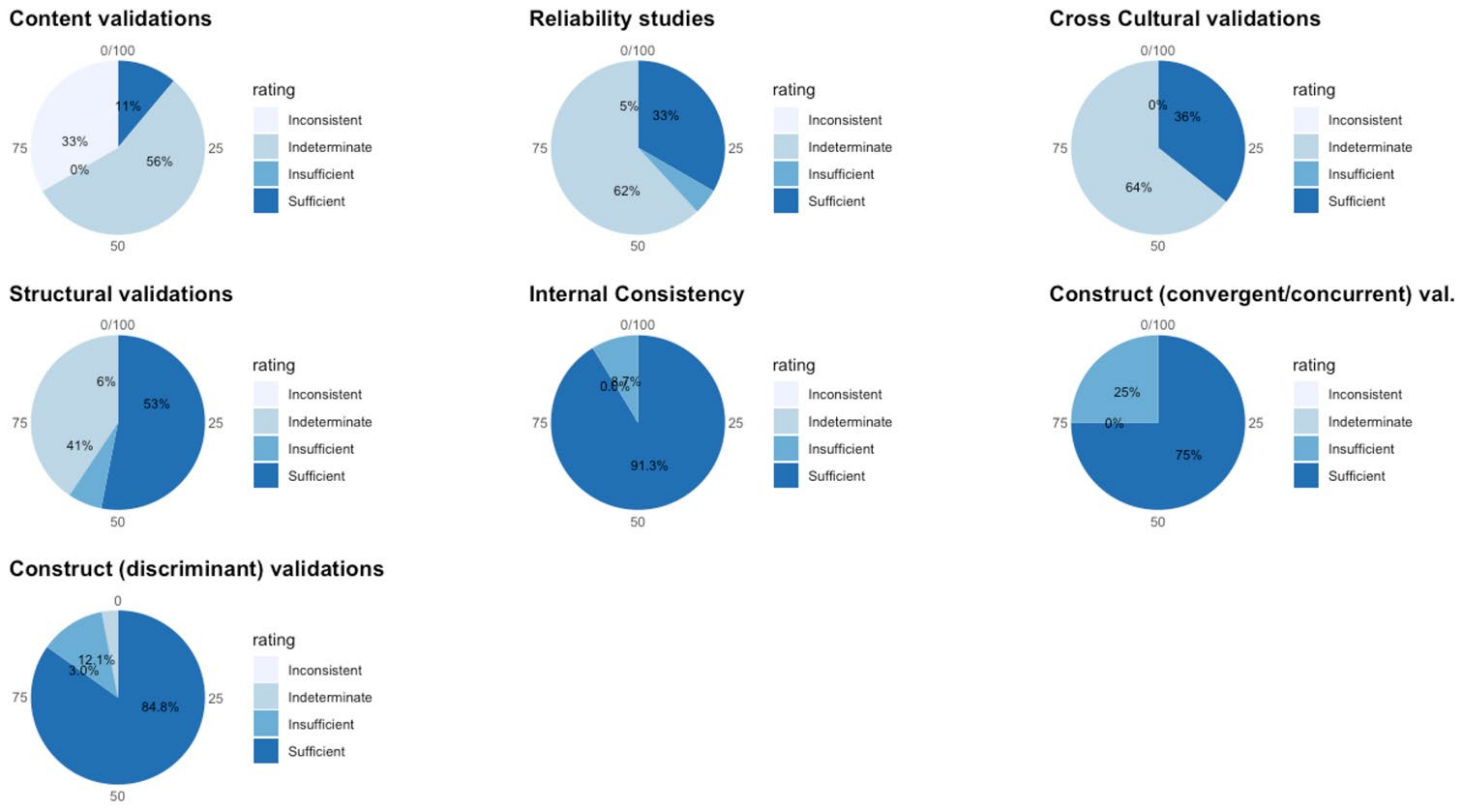
Summary of Results Ratings for Measurement Properties

**Fig. 4:**
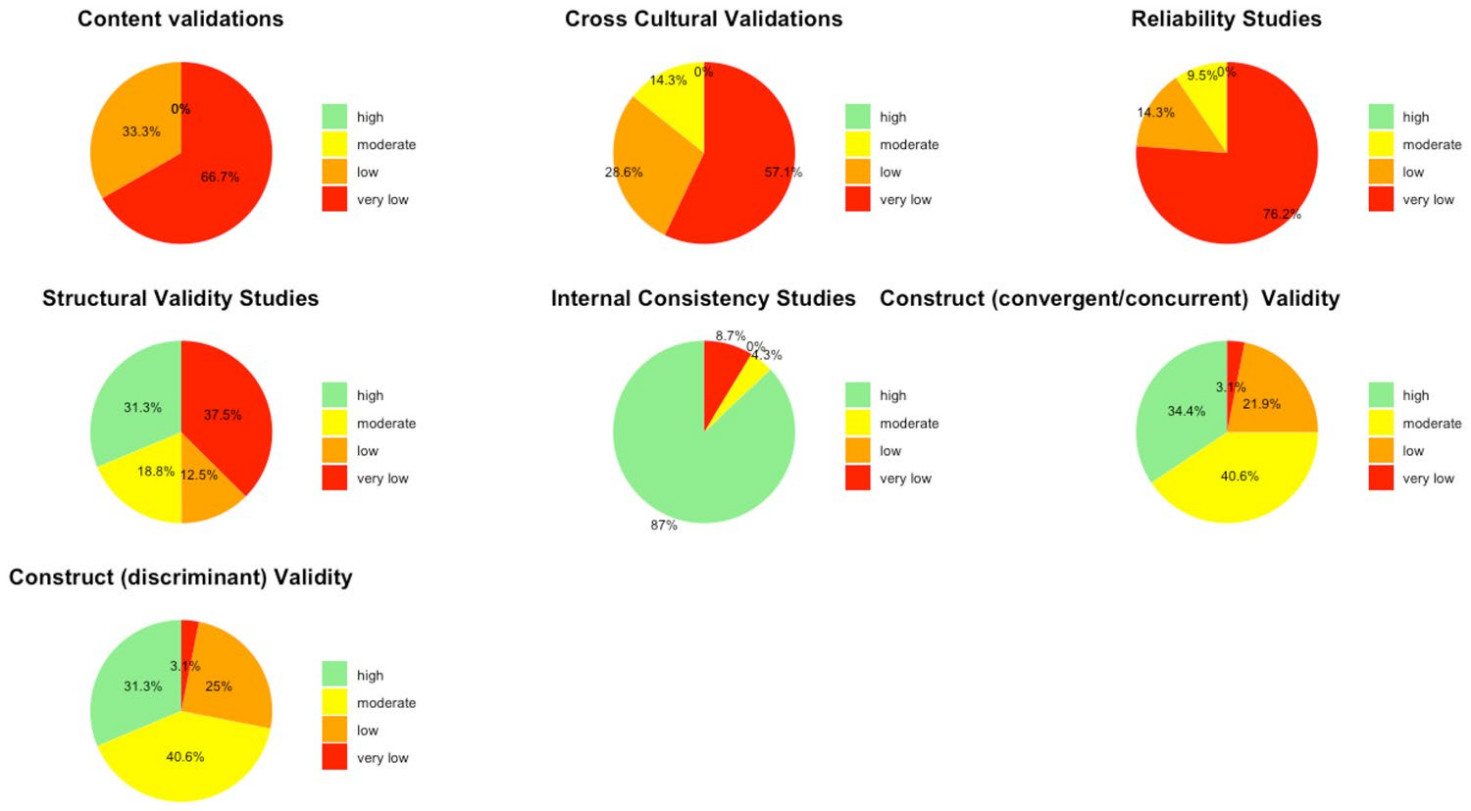
Summary of Quality of Evidence (GRADE) Ratings for Measurement Properties

In this systematic review, 40 unique tools coming in 49 version/variants were identified as having been either developed or adapted/validated for use among children in LAMIC countries. Figures 3 and 4 below provide a snapshot summary of the overall results of this critical appraisal in pie chart format. Figure 3 shows the overall summary of the results ratings of the various measurement properties, while Fig. 4 shows the summary of the overall GRADE ratings of the quality of evidence for the various measurement properties. None of these tools showed full validation (i.e., validation in all domains assessed) in LAMICs. Only 11% of adaptation/development studies showed “sufficient” content validation, while 33% showed sufficient results for reliability and 36% did same for cross cultural validity. However, for internal consistency, structural, convergent and discriminant validities results were good at 91.3%, 53%, 75% and 84% “sufficient” results respectively. When it came to the quality of evidence as well (see Fig. 4), the pattern of results was similar with 100% of content validation studies, 86% of cross-cultural validations and 90.5% of reliability studies being of either low or very low quality. Internal consistency was generally of high quality (87%), with 50.1% of structural validity, 75% of convergent/concurrent studies and 71.8% of discriminant validity studies showing either high or moderate quality of evidence supporting their results.

Going into the specific instruments (see Table 8), the BRIEF (in its various versions) (Gioia et al., 2000), Wisconsin Card Sorting Test (WCST) (Berg, 1948), Go/No-go (GNG) (Luria, 1973) and the Rey-Osterrieth complex figure (ROCF) (Rey, 1941) had the most validations undertaken, both in terms of the variety of validations and the number of studies. None of them though had either ‘high’ or even ‘moderate’ quality evidence for ‘sufficient’ psychometric properties across all the measured psychometric domains. The BRIEF (in all its versions) showed high quality of evidence for ‘sufficient’ internal consistency, high quality evidence for ‘sufficient’ construct validity (for BRIEF- pre-school version) and moderate to low quality evidence for sufficient construct validity (mostly convergent validity) for BRIEF-parents and BRIEF-teachers respectively. The WCST showed moderate quality evidence for ‘sufficient’ internal consistency and low-quality evidence for ‘sufficient’ construct validity across several studies, while the ROCF showed high quality evidence for ‘sufficient’ construct validity (discriminant validity). The GNG showed high quality evidence for both ‘sufficient’ structural validity and ‘sufficient’ internal consistency. For cross-cultural validity, only two instruments had moderate quality evidence for a ‘sufficient’ rating: GNG and the Behavioural Assessment System for Children (BASC) (Reynolds & Kamphaus, 1992). They both also showed high quality evidence for sufficient structural validity and internal consistency. However, BASC showed low quality evidence for sufficient construct validity in the countries of interest.

For adaptive functioning, the most assessed tools were the Vineland Adaptive Behaviour Scales (VABS) (Sparrow et al., 1984, 2005) and the Child Function Impairment Rating Scale (CFIRS) (Tol et al., 2011), a newly developed tool from Indonesia. Of these two, while both had high quality evidence for ‘sufficient’ internal consistency, VABS showed low-quality evidence for ‘insufficient’ structural validity, and indeterminate cross-cultural validity. The CFIRS showed low quality evidence for sufficient construct validity but reported no formal assessment of comprehensibility and comprehensiveness among subjects during tool development (hence was rated ‘indeterminate’ in both) with methodological shortcomings in relevance assessment (hence rated ‘insufficient’).

Table 9 shows a summary of the results of the critical appraisal for content validity. First few columns show results of **individual studies** content validation studies, with the “overall rating” column showing the **synthesis of results** for all content validation studies pertaining to **an instrument**, and the colour coding showing the GRADE rating for quality of evidence. For details of the reasons behind each results rating, see comments on ‘Summary Content validity’ in supplemental material; and for Risk of Bias of content validation for individual instrument development studies, see COSMIN box 1, while that for content validation of adaptation studies is in COSMIN box 2 in the supplemental material.

Here, 16 unique tools coming in 18 version/variants had either instrument development studies (i.e., brand new tools developed for the countries of interest) or adaptation studies reporting on their content validity in those countries, which were critically appraised. None of these tools showed full content validity (i.e., ‘sufficient’ rating of high or moderate quality evidence in all three sub-domains of RELEVANCE, COMPREHENSIBILITY and COMPREHENSIVENESS) in the LAMICs considered. Of these, only the BRIEF (pre-school version) (Isquith et al., 2004) and the VABS (Sparrow et al., 1984, 2005) had studies showing ‘sufficient’ content validity but of very low quality of evidence, with all the others showing either ‘indeterminate’ (?) or ‘inconsistent’ ( ±) content validity results of either low or very low quality of evidence. For several of the legacy tests such as the Wisconsin Card Sorting Test (WCST) (Berg, 1948), Go/No-go (GNG) (Luria, 1973) and the Rey-Osterrieth complex figure (ROCF) (Rey, 1941), the original development studies could not be obtained to be critically appraised. Therefore we estimated their probable score based on the best case scenario of their likelihood of fulfilling (or failing to fulfil) the COSMIN criteria for development studies, which was used in estimating their overall content validation score (while factoring in the score of the adaptation studies that were eligible as described in *Synthesis of Results* section of Methodology, and in COSMIN guideline pages 62–63), with reasons provided for their estimated score.

## Discussion

This systematic review was carried out to critically appraise the validation and adaptation studies that had been conducted for use of executive and adaptive functioning tools among children and adolescents in LAMICs, to make recommendations for clinical use and for further research in such cross-cultural contexts.

Summary of the evidence: The General State of the Evidence Available.

The first noteworthy observation is that there is an obvious lack of validation studies for executive functioning (EF) and adaptive functioning (AF) instruments in LAMICs, period. The many ‘potholes’ in the summary tables of Tables 8 and 9 is ample evidence of this lack of validity studies. Further, even where the evidence exists, the quality of evidence leaves much to be desired. Particularly concerning were the quality of evidence for content validation, cross-cultural validation, reliability and (to a lesser extent) structural validation studies, where most of these types of studies were of either ‘low’ or ‘very low’ quality as shown in Figs. 3 and 4.

For cross-cultural validation studies, only 5 out of 14 (35%) showed ‘sufficient’ cross cultural validity (see Fig. 3), and even then, the quality of evidence for 12 out of 14 (86%) was ‘low’ or ‘very low’ (see Fig. 4). One of the major methodological problems that led to this poor result was that a vast majority of papers purporting to do a cross-cultural validation failed to use an appropriate approach to compare the two groups (comparing original culture to target cultural context) such as Multi-group Confirmatory Factor Analysis (MGCFA) with most opting for a Principal Component Analysis (PCA), or even ANOVAs and *student t test* approaches. In order to adequately ascertain measurement invariance of a tool in one cultural context as compared to its use in its original context, one must assess how each item in the tool functions in the target population- whether the items achieved scalar invariance, metric invariance and configural invariance- to be able to confidently conclude that the tool functions in the same way in the target population as it does in the original population in which it was developed (Fischer & Karl, 2019). Only an appropriate method such as MGCFA can provide this information. A PCA for example, based on its underlying assumption of only common variance and no unique variance among items, will only be useful as a data reduction tool that can essentially “boil down” a complex set of variables to an essential set of composite variables (i.e. reduce the number of necessary items in a tool), and not to uncover the latent constructs underlying the variables or to ascertain how items behave in another population. Therefore, use of PCA for cross-cultural validation is considered an inappropriate methodology, and was the major reason for most of the cross-cultural validation studies being rated so poorly, as per COSMIN criteria.

Reliability studies also had a poor showing. Only 6 out of 21 (29%) showed ‘sufficient’ reliability, and even then, the quality of evidence for 19 out of 21 (90%) was ‘low’ or ‘very low’. The reason for this poor showing for many of the studies was either time interval between assessments not stated, or too long an interval (whether for test–retest or inter-rater reliability), where many studies went well beyond the recommended (by COSMIN) 2 week-period (some studies as long as 48 – 96 weeks between assessments), taking too long a time interval to be sure that the underlying construct (e.g. EF) had not actually changed in reality, especially considering that EF does change with age. A second reason was an inappropriate method of data analysis, where some papers used a Pearson’s correlation to calculate reliability without any evidence provided that no systematic change had occurred (since Pearson’s does not take systematic error into account), rather than using the more appropriate Intra Class Coefficient (ICC) (which does take systematic errors into account) and describing the model of ICC used.

For content validity, only 2 out of 16 studies (13%) had ‘sufficient’ content validity with all of them showing either ‘low’ or ‘very low’ quality of evidence. The commonest reasons for this were that many adaptation or instrument development studies did not involve the target audience in either concept elicitation (for instrument development studies) or the determination of relevance, comprehensiveness or comprehensibility (for adaptation studies), with most relying heavily on “expert panel” input for these things. Given the modern sweep towards “co-production” methods, this “expert dominated” synthesis cannot continue to be encouraged. Also, most did not report on enough detail of the pilot studies to allow for adequate assessment of methodology, hence the many ‘indeterminate’ ratings of content validity (according to COSMIN, where not enough information is provided to make a judgement one way or another whether evidence is ‘sufficient’ or not, it is recommended to make a rating of ‘indeterminate’). Further, in a few cases where either legacy instruments (such as WCST and ROCF) or some proprietary instruments were involved, it was not always possible to obtain the original development studies if they were not freely available online (some proprietary instruments only published their development study in their instrument manuals which need to be purchases along with the tools), and therefore an estimation rating had to be made for the development tools based on information that was publicly available, assuming a best-case scenario each time.

Having noted the above, the best performing tools for use among children in LAMICs (judging by quality of evidence for psychometric properties) were BRIEF for executive function (EF) and VABS for adaptive function (AF). For EFs, the main weakness of the close contenders to the BRIEF- the legacy instruments/tasks of WCST, GNG and ROCF- was their lack of comprehensiveness in assessing ALL domains of EF as currently widely accepted. The BRIEF (in all its various versions) showed a respectable coverage of almost all the domains of validity that were assessed with positive results in a good number of them: there was high quality evidence for ‘sufficient’ internal consistency (seen in 7 studies across all versions of BRIEFs); ‘sufficient’ construct validity (mostly convergent and some discriminant validity) whose quality of evidence ranged from low to high (seen in 5 studies across 3 out of 4 versions of BRIEFs); and moderate to low quality evidence for ‘sufficient’ construct validity (mostly convergent validity) for BRIEF-parents and BRIEF-teachers versions respectively. However evidence for good content validity, structural validity and cross-cultural validity of the BRIEFs among children in LAMIC was lacking: there was very low quality evidence for one 'indeterminate' rating of content validity (parent version) and one 'sufficient' content validity (pre-school version) seen in 2 studies; low to very low quality evidence for mostly 'indeterminate' structural validity seen in 6 studies across all versions of BRIEF (except for one study showing low quality evidence of 'sufficient' structural validity of BRIEF-teacher version); and low quality evidence for 'indeterminate' cross-cultural validity (seen in 2 studies but only for 2 versions- parent and pre-school).

Having noted the above, an honourable mention is the TEXI/CHEXI tool, which although a relatively new tool with fewer studies conducted in LAMICs showed remarkably good results with decent quality of evidence: high quality evidence for ‘sufficient’ internal consistency (seen in 2 studies across all versions), low to high quality evidence for ‘sufficient’ construct validity (seen in 2 studies across all versions), moderate quality evidence for ‘sufficient’ structural validity (2 studies across all versions) and low quality evidence for ‘sufficient’ reliability (seen in 1 study for TEXI). The EFICA tool- developed in Brazil- was also noteworthy because although there was only 1 study evaluating it, that single study produced high quality evidence of sufficient structural validity, internal consistency and (for the parent version only) construct validity (discriminant validity) across its 2 versions- parent and teachers. Both of these other tools though did not have good evidence for content validity in their reported studies. Glaringly missing in all EF instruments though was any tool that explicitly assesses the domain of meta-cognition, but this is probably due to the continuing evolution and refinement of the concept even in Western thought.

For adaptive functioning (AF), VABS was the most validated as earlier mentioned. The main weakness of the closest contender, the CFIRS, was the fact that outside of its home country of Indonesia it had not been validated in a cross-cultural setting, which is understandable given its relatively newer status. As has been noted elsewhere in the literature, adaptive behaviour and expectations of daily living skills and socialization are culturally dependent, hence local input into the appropriate types of questions to be asked in adaptive behavior assessment is crucial (Semrud-Clikeman et al., 2017). The VABS (in all its editions) also showed a decent coverage of almost all domains of validity evaluated with positive results: high quality evidence of sufficient internal consistency (seen in 2 studies) and construct validity (backed by 2 studies); low quality evidence for ‘sufficient’ reliability (backed by 2 studies); and very low-quality evidence for sufficient content validity (backed by an ‘inadequate’ adaptation study and an ‘inadequate’ development study). However, results for cross-cultural validity and structural validity were not the best: there was low quality evidence for an ‘indeterminate’ rating of cross culturally validity (2 studies); and very low-quality evidence of insufficient structural validity (backed by 1 study).

### Evidence from High-Income Countries

Evidence from HICs indicate that assessments of EF and adaptive behavior form a very significant proportion of all assessments performed by clinical psychologists and neuropsychologists with approximately 50% of clinical psychologists practicing in this area (Camara et al., 2000) which indicates its importance. In this nationally representative survey of 1002 neuropsychologists and 1500 clinical psychologists, the most frequently used EF instruments were such legacy tests as Trail making test (TMT) and WCST while that for adaptive behaviour was the VABS (Camara et al., 2000), which was confirmed by a broader-based and more recent follow up survey among 2004 north American neuropsychologists (Rabin et al., 2005), with the ROCF and NEPSY also featuring more prominently in this survey. To provide some context though, it is worth noting that these large surveys are rather dated as the data were collected over 20 years ago, hence would not have included many of the instruments included in this review which were developed in the last 20 years.

Interestingly, in a similar recently published systematic review of EF performance-based instruments used within the context of Occupational therapy (which incidentally also used the COSMIN manual for assessing risk of bias, and which was not limited to LMICs), the following instruments, none of which have been adapted for use in LAMICs hence failed to show up in our review, were all found to have a “low” GRADE rating of quality of evidence: the Behavioural Assessment of the Dysexecutive Syndrome for Children, Children’s Cooking Task, Children’s Kitchen Task Assessment, Do-Eat, and Preschool Executive Task Assessment (Gomez et al., 2021). Another such systematic review performed by researchers from Italy (also using COSMIN, and also not limited to LAMICs, but considering a much limited array of databases compared to this present study) also identified 19 EF tools which scored poorly on their methodological rigour, but also found the BRIEF (2 of its variants) to have the best risk of bias rating, noting that it particularly did well in internal consistency, but reported inadequate indices for reliability and other measurement properties (Berardi et al., 2021). This study also noted the relatively good performance of another test not included in our review (for lack of adaptation in LMIC)- the Lion Game (Van de Weijer-Bergsma et al., 2014) developed for study of visuo-spatial working memory in Dutch. Finally another paper reviewing EF tools used in Brazil was also found (Guerra et al., 2020) however no critical appraisal was performed in this paper. These findings from the wider literature therefore largely confirm the findings in this systematic review.

### Cross Cultural Considerations

Since one of the desired outcomes of this paper is to be able to make recommendations of assessment tools for use in LAMICs, an important consideration for the authors was which of these instruments had adequate content validity and cross-cultural validity in LAMICs. The importance of adequate cultural adaptation or development of instruments in accurately measuring such psychological constructs cannot be overstated. This very point was made by Geisinger in his seminal paper on cross-cultural normative assessment (Geisinger, 1994) when he highlighted the importance of going beyond simple language translation and considering cultural backgrounds during adaptation of instruments among sub-populations in the US. Concept formation is goal-directed. Which means, if the socio-cultural milieu within which a child is placed does not place certain types of goals before him/her, he/she will not form concepts to solve the problems standing as obstacles to that goal, a point echoed by scholars across the decades from Vygotsky first in the 1930’s (Vygotsky, 1980) through to Ardila (1995), and Nell (1999) more recently while discussing the work of Luria (1973). For a word to have any meaningful meaning, it must represent a concept that is widely shared with the speakers to whom the communication is directed. Or put in the words of Vygotsky: “This is why certain thoughts cannot be communicated to children even if they are familiar with the necessary words” (Vygotsky, 1980). Any task or set of questions placed before this child (or caregiver) that has not been rigorously determined to be within the conceptual understanding of said child, therefore, is bound to fail in assessing what it purports to assess without a proper rigorous cultural (and conceptual) adaptation. This type of “cultural specificity” of conceptualization has been well documented in the literature in studies from across the world, for example by Venter in the context of the acquisition of Piagetian tasks in cognitive development (Venter, 2000).

Unsurprisingly, the EF instruments that performed best in cross-cultural settings were legacy performance-based tools such as the GNG (moderate quality evidence of sufficient cross-cultural validity) and Tower Test (low quality evidence of sufficient cross-cultural validity), with the one interview-based instrument that did reasonably well being the BASC (moderate quality evidence of sufficient cross-cultural validity). Of these, the only instrument for which a formal adaptation was reported was the GNG, which showed low quality evidence for 'inconsistent' rating of content validity. Further, it is reported elsewhere in the literature from LAMICs that a version of the GNG (the knock tap test) should be used with extreme caution because of poor reliability (Semrud-Clikeman et al., 2017).

An important implication of these finding is that potentially performance-based tasks/tools are perhaps most easily translatable across different cross-cultural contexts to produce consistent results across different population groups. An obvious shortcoming of this though is the issue of most of these existing (legacy) tasks lacking comprehensiveness in terms of our current understanding of the various domains of EF. A potential solution to this dilemma might be the development of more comprehensive, culturally appropriate performance-based measures of EF which tap into almost all domains of EF. These could either be universally adaptable age-appropriate tasks- such as cooking or running errands- or more regionally peculiar tasks- such as fetching water, playing of particular local games or occupational activities like farming- if they could potentially be calibrated to assess all domains of EF and AF as we currently understand them.

### Tool Recommendations

One of the important goals of this paper was to be able to make evidence-backed recommendations for appropriate tools for EF and AF among children and adolescents in LAMICs. To fulfil this goal, one potential approach could have been to make recommendations for the best tools for each domain of EF and AF and discuss reasons why accordingly. However, apart from how bulky this approach would have made this paper, again we were guided by the insightful observation of Vygotsky on the matter of assessing only the constituent parts of a complex whole thus (Vygotsky, 1980): rather than assessing EF and AF only in terms of their constituent domains (as some EF tools for example appear to do), we chose to treat EF and AF as complex wholes because of their intricate inter-linkages and their conceptual unity in our current modern understanding of them.

According to the COSMIN guidelines (as well as the Standards for Educational and Psychological Testing (American Educational Research Association, American Psychological Association, & National Council on Measurement in Education, 2018)), content validation is the most important validation as every other psychometric property of the instrument in question hinges on the items/content making sense in the population to be measured (with respect to the construct of interest- as understood by the population) (Terwee et al., 2018a, b). That notwithstanding, a word of caution from the manual about content validation is in order: “The content validity of an [instrument] may be different when using the [instrument] for measuring different constructs, in different populations, or in different contexts of use. Researchers do not validate an instrument but rather the **application** of it" (Terwee et al., 2018a, b) (emphasis ours). Considering all this and considering their overall performance in the other domains and measurement properties of validity, the BRIEF and VABS would again merit some measured recommendation for use among children in LAMICs as they both have studies showing 'sufficient' content validity, even though of very low-quality evidence. All other instruments evaluated showed either 'inconsistent' or 'indeterminate' content validity of either 'low' or 'very low' quality. Further, all of those others showed slightly better quality of evidence ('low' as opposed to 'very low') only because their instrument development studies could not be obtained and hence a best-case-scenario estimation of their development study quality of evidence (i.e., assuming the best, given available evidence) was a 'doubtful' overall rating, giving them 'low' (as opposed to 'very low') quality of evidence.

Having said that, one of the issues with the BRIEF in Western countries is the lack of positive correlation with direct measures of executive functioning (Semrud-Clikeman et al., 2017). The implications of this is that the BRIEF may reflect a child’s inability to perform these skills in day-to-day life even though individual measures of executive functioning may show that the child can perform the task in a structured, one-to-one setting (Semrud-Clikeman et al., 2017). But in its defense, this problem of a disconnect between legacy “experimental” tests of EF and the “real life situations” at home is what prompted the developers of the BRIEF to come out with their “ecologically valid” assessment instrument in the first place (Gioia et al., 2000).

Other reports in the literature largely support our findings in this review. In the scoping review paper for NDD assessment tools for children in LAMICs, among the EF tools they reported as having been used in LAMICs were the NEPSY (in South Africa, Zambia, Romania and Iran) where they found that strong language skills are needed for success, even for English-speaking subjects; the Behaviour Rating Inventory of Executive Function (BRIEF) in Uganda, and the Wisconsin Card Sorting Test (WCST) in Uganda where they reported poor reliability due to the nature of the test (Semrud-Clikeman et al., 2017). For adaptive functioning, this paper reported that the Vineland Adaptive Behavior Scales (VABS) (Sparrow et al., 2005) as the commonest reported in LAMICs, which has been used successfully in South Africa with families with HIV (Allen et al., 2014). Semrud-Clikeman and colleagues did go ahead to finally recommend selected subtests of the NEPSY-II for testing executive function, and the VABS and the IBAS for testing adaptive behavior (with limitations) which although not backed by a rigorous critical appraisal of the evidence, nonetheless largely supports our recommendations.

### Future Directions

One may wonder though, after such an apparently less-than-ideal review, if there is hope for a more universally applicable assessment of executive and adaptive functions. To this, we offer the well-known aphorism: the absence of evidence is not the evidence of absence; that is to say, more evidence needs to be collected before utterly rejecting many of these tools. To be fair to most of these tests, the COSMIN criteria being a recent development is rather biased in favor of tools developed along the contemporary principles of co-production and patient/stakeholder involvement in research design and implementation, which is a relatively recent phenomenon. Therefore, for several of these legacy instruments (being developed 20 or more years ago when such principles were not yet contemporary), it is not too surprising that the COSMIN criteria penalized them for this. However, if LAMICs are to keep abreast with current best practices, it is imperative that researchers and clinicians are made aware of how available existing tools hold up against these best practices, and the standards that the scientific community will expect in adapting or developing tools for local use.

Content validation, being the most important measurement property of an instrument upon which all other properties are hinged (Terwee et al., 2018a, b), bears singling out for comment. BRIEF and VABS showed the most promise for use in LAMICs, but can have better content validation studies and cross-cultural studies undertaken. For example, in the adaptation study of the BRIEF (parents) by Chernoff et al*.* (Chernoff et al., 2018), no cognitive interviewing (Jobe & Mingay, 1989) was done at all which is a fatal flaw in ensuring content validity, while in that of the BRIEF (pre-school) by Diaz et al. (Diaz & Anacona, 2017) a form of cognitive interviewing was done but parents were apparently not specifically asked about the relevance of items in their sociocultural experience. This aspect of adaptation of western tools for cross-cultural use needs to be improved by researchers, with more involvement of parents, adolescents, and teachers in a cognitive interviewing approach to assess relevance and comprehensibility of items and instructions (and due modification where appropriate) going forward.

Finally, researchers in LMICs might want to consider more performance-based task assessment tools that are ecologically based (such as the Children’s Cooking Task (Chevignard et al., 2010)) and are culturally appropriate for future adaptation. As was noted above under the “cross cultural considerations” sub-section, the best performing assessment tools in cross cultural contexts were performance-based tools such as the GNG and Tower Test. Some of these tasks (with some minor cultural fine-tuning of course) might find greater universal applicability (simple cooking tasks for example are likely to find applicability in almost all cultures around the world) and might represent a potentially more universal basis of comparison across various cultures than the various questionnaire-based assessment tools. They are also likely to better solve the problems of subjectivity in interpretation of items by respondents and the need for literacy (a particularly acute problem in LAMICs) in the use of questionnaire-based assessment instruments.

### Strengths and Limitations of this Study

This systematic review was conducted according to the rigorous guidelines of PRISMA checklist for reporting Systematic Reviews (Liberati et al., 2009), with the risk of bias assessment conducted following the high standards of both the COSMIN guidelines for evaluation of content validation studies (Terwee et al., 2018a, b) (for content validation of adaptation and development studies) and the ‘COSMIN Manual for Systematic Reviews of PROMs’ (Mokkink et al., 2018a) (for all other validations). The complex and varied sources of evidence were qualitatively summarized in an easy-to-digest format, while giving the benefit of doubt where it was reasonable to do so, to make some evidence-based recommendations where they could be made (and to stay silent where evidence was simply not enough to conclude one way or the other). We also searched an exceptionally large number of databases (14 in all, including grey literature), and included as many non-English papers as we could translate in this study.

Unfortunately, there were some noteworthy limitations to our study. Firstly, not all eligible abstracts could be obtained or translated. These have been listed in Appendix II. Secondly, The COSMIN guidelines that formed the backbone of the risk of bias assessment of methodology was primarily designed for use for “patient reported outcome measures" or PROMs- i.e., for interview-based questionnaires. The criteria therefore had to be adapted by authors for the evaluation of performance-based tasks/instruments, in places where the questionnaire-targeted criteria would not be appropriate for such performance-based instruments. However, we have endeavoured to be as transparent as possible where these adaptations were made and why and are of the opinion that these adaptations did not affect the overall impression of the quality of evidence for each performance-based task as most COSMIN criteria could be reasonably applied to such tasks (as well as to questionnaires). Thirdly, we did not assess practical considerations like copyright issues and cost surrounding use of these tools, which are all important and relevant since these can all affect the availability and use of such tools particularly for resource-poor LAMICs. Finally, in a few cases where either legacy instruments (such as WCST and ROCF) or some proprietary instruments were involved, it was not possible to obtain the original development studies if they were not freely available online (some proprietary instruments only published their development in their instrument manuals which need to be purchases along with the tools), and therefore an estimation rating had to be made for the development tools based on information that was publicly available, assuming a best-case scenario each time.

## Conclusion

To the best of our knowledge, this is the first systematic review of EF and AF tools focused on children in LAMICs of the scope and depth that we have done. In it we sought to critically appraise the kind and quality of evidence available rigorously for the psychometric soundness of instruments developed or adapted for use in LAMIC and made recommendations based on the evidence we found. From this careful evaluation, the BRIEF and the VABS appeared to have the best evidence for use in this context. However, we must stress that the conclusions or findings from this review are not absolute. They are limited to findings of evidence (or lack thereof) as of the time of research, and (more importantly) for the country-settings of interest. Therefore, our recommendations notwithstanding, it is not to categorically say that that the other instruments are inadequate for use, or that good evidence for each of these (other) instruments cannot be generated in the future, or that even now there is not good evidence for their use in HICs or other settings (other than LAMICs). Therefore, interpretation of results must be limited only to current evidence available as of time of publication and to the specified country-contexts evaluated.

### Supplementary Information

Below is the link to the electronic supplementary material.

Supplementary file1 (XLSX 670 KB)

## Appendices

### Appendix I: Search Strategy for MEDLINE

1. "executive function*".ti,ab.
2. “executive dysfunction*”.ti,ab.
3. “dysexecutive syndrome”.ti,ab.
4. "frontal lobe* function*".ti,ab.
5. "frontal lobe* dysfunction*".ti,ab.
6. "function* of the frontal lobe*".ti,ab.
7. “frontal lobe* syndrome”.ti,ab.
8. "frontal lobe* damage".ti,ab.
9. “prefrontal cortical damage”.ti,ab.
10. “prefrontal damage”.ti,ab.
11. “prefrontal cortical dysfunction*”.ti,ab.
12. “prefrontal dysfunction*”.ti,ab.
13. “prefrontal cortical function*”.ti,ab.
14. “prefrontal function*”.ti,ab.
15. “adaptive function*”.ti,ab.
16. “adaptive function* impair*”.ti,ab.
17. “activities of daily living”/
18. “daily living”.ti,ab.
19. “ADL”.ti,ab.
20. or/1-14
21. or/15-19
22. “children”.ti,ab.
23. 21 and 22
24. “valid*".ti,ab.
25. “reliab*”.ti,ab.
26. “unreliab*”.ti,ab.
27. standard*”.ti,ab.
28. “norm*”*".ti,ab.
29. “reproducibl*”.ti,ab.
30. “replica*”.ti,ab.
31. “coefficient of variation”.ti,ab.
32. “internal consistency”.ti,ab.
33. “responsive*”.ti,ab.
34. “assessment tool*”.ti,ab.
35. “assessment instrument*”.ti,ab.
36. “outcome assessment”.ti,ab.
37. “outcome measure*”.ti,ab.
38. “clinimetr*”.ti,ab.
39. “clinometr*”.ti,ab.
40. “cross-cultural”.ti,ab.
41. "reproducibility of results"/
42. "sensitivity and specificity"/
43. behavior rating scale/
44. neuropsychological tests/
45. psychometrics/
46. discriminant analysis/
47. Or/24-46
48. Developing Countr***.sh,kf.**
49. (low adj3 middle adj3 countr*)**.ti,ab.**
50. (lmic or lmics or third world or lami countr*)**.ti,ab.**
51. transitional countr***.ti,ab.**
52. ((developing or less* developed or under developed or underdeveloped or middle income or low* income or underserved or under served or deprived or poor*) adj (countr* or nation? or population? or world))**.ti,ab.**
53. ((developing or less* developed or under developed or underdeveloped or middle income or low* income) adj (economy or economies))**.ti,ab.**
54. (low* adj (gdp or gnp or gross domestic or gross national))**.ti,ab.**
55. (Africa or Asia or Caribbean or West Indies or South America or Latin America or Central America)**.hw,kf,ti,ab,cp.**
56. (Afghanistan or Albania or Algeria or Angola or Antigua or Barbuda or Argentina or Armenia or Armenian or Aruba or Azerbaijan or Bahrain or Bangladesh or Barbados or Benin or Byelarus or Byelorussian or Belarus or Belorussian or Belorussia or Belize or Bhutan or Bolivia or Bosnia or Herzegovina or Hercegovina or Botswana or Brasil or Brazil or Bulgaria or Burkina Faso or Burkina Fasso or Upper Volta or Burundi or Urundi or Cambodia or Khmer Republic or Kampuchea or Cameroon or Cameroons or Cameron or Camerons or Cape Verde or Central African Republic or Chad or Chile or China or Colombia or Comoros or Comoro Islands or Comores or Mayotte or Congo or Zaire or Costa Rica or Cote d'Ivoire or Ivory Coast or Croatia or Cuba or Cyprus or Czechoslovakia or Czech Republic or Slovakia or Slovak Republic or Djibouti or French Somaliland or Dominica or Dominican Republic or East Timor or East Timur or Timor Leste or Ecuador or Egypt or United Arab Republic or El Salvador or Eritrea or Estonia or Ethiopia or Fiji or Gabon or Gabonese Republic or Gambia or Gaza or Georgia Republic or Georgian Republic or Ghana or Gold Coast or Greece or Grenada or Guatemala or Guinea or Guam or Guiana or Guyana or Haiti or Honduras or Hungary or India or Maldives or Indonesia or Iran or Iraq or Isle of Man or Jamaica or Jordan or Kazakhstan or Kazakh or Kenya or Kiribati or Korea or Kosovo or Kyrgyzstan or Kirghizia or Kyrgyz Republic or Kirghiz or Kirgizstan or Lao PDR or Laos or Latvia or Lebanon or Lesotho or Basutoland or Liberia or Libya or Lithuania or Macedonia or Madagascar or Malagasy Republic or Malaysia or Malaya or Malay or Sabah or Sarawak or Malawi or Nyasaland or Mali or Malta or Marshall Islands or Mauritania or Mauritius or Agalega Islands or Mexico or Micronesia or Middle East or Moldova or Moldovia or Moldovian or Mongolia or Montenegro or Morocco or Ifni or Mozambique or Myanmar or Myanma or Burma or Namibia or Nepal or Netherlands Antilles or New Caledonia or Nicaragua or Niger or Nigeria or Northern Mariana Islands or Oman or Muscat or Pakistan or Palau or Palestine or Panama or Paraguay or Peru or Philippines or Philipines or Phillipines or Phillippines or Poland or Portugal or Puerto Rico or Romania or Rumania or Roumania or Russia or Russian or Rwanda or Ruanda or Saint Kitts or St Kitts or Nevis or Saint Lucia or St Lucia or Saint Vincent or St Vincent or Grenadines or Samoa or Samoan Islands or Navigator Island or Navigator Islands or Sao Tome or Saudi Arabia or Senegal or Serbia or Montenegro or Seychelles or Sierra Leone or Slovenia or Sri Lanka or Ceylon or Solomon Islands or Somalia or South Africa or Sudan or Suriname or Surinam or Swaziland or Syria or Tajikistan or Tadzhikistan or Tadjikistan or Tadzhik or Tanzania or Thailand or Togo or Togolese Republic or Tonga or Trinidad or Tobago or Tunisia or Turkey or Turkmenistan or Turkmen or Uganda or Ukraine or Uruguay or USSR or Soviet Union or Union of Soviet Socialist Republics or Uzbekistan or Uzbek or Vanuatu or New Hebrides or Venezuela or Vietnam or Viet Nam or West Bank or Yemen or Yugoslavia or Zambia or Zimbabwe or Rhodesia)**.hw,kf,ti,ab,cp.**
57. Or/48-57
58. 20 and 23 and 47 and 57

NB: for search items 48 – 56, a special search filter for ‘developing countries’ authored by the Cochrane Library (Cochrane Library, 2012) was used verbatim.

### Appendix II: Untranslated and/or Unavailable but Potentially Eligible Non-English Articles

1. Zhang Q. 2003 Development of Adaptive Skill Rating Scale for school age children: *only Chinese PDF available*
2. Yao S. 1999 Development of the Adaptive Skill Rating Scale for Young Children (ASRSYC): *online version not available*
3. Rosselli-Cock M. 2004 Neuropsychological assessment of children: A test battery for children between 5 and 16 years of age. A Colombian normative study: *online version not available*
4. Pereira A. 2018 Executive functions in childhood: Assessment and preliminary normative data for Portuguese preschoolers: *study conducted in Brazil but online pdf version not available *
5. Du Q. 2010 Reliability and validity of the Behavior Rating Inventory of Executive Function-Adult Version Self-Report Form in China: *pdf version not available online*
6. Singh S. 2019 A comparative study of vineland adaptive behavior scale ii and vineland social maturity scale on children and adolescents with intellectual disability: *online version not readily available*
7. Pawlowski J. 2014 Evidences of construct validity of the NEUPSILIN using confirmatory factorial analysis: *only Portuguese pdf available, was unable to get translator*
8. Mashhadi A. 2014 Psychometric properties of the Behavior Rating Inventory of Executive Functioning-Preschool Version (Teacher Form): *only Persian version of pdf available, hence was difficult to translate using google translate. *
9. Pawlowski J. 2013 Reliability of the Brief Neuropsychological Assessment Instrument Neupsilin: *only Portuguese pdf version available, hence difficult to translate*
10. Ramos-Galarza C. 2019 EFECO Scale for Assessing Executive Functions in Self-Report Format: *for this study done in Ecuador, only Spanish version in pdf was available, hence difficult to translate*
11. Butman J. 2000 Spanish verbal fluency. Normative data in Argentina: *could not obtain full article online. *
12. Barreyro J. 2015 Validity and Reliability of the Running Memory Span: *only Spanish pdf version available online. *
13. Roselli Cock M. 2005 Neuropsychological Assessment of Children: a test battery for children between 5 and 16 years of age. A Colombian normative study: *only Spanish pdf version available online. *
14. Qian Y. 2011 Reliability and validity of the Chinese version of Weiss Functional Impairment Scale-Parent form for school age children: *no pdf version available at all online*
15. Dadsetan P. 2010 Kindergarten Inventory of Social/Emotional Tendencies: A cross-validation study: *no pdf available at all online*
16. Shi-jie Z. 2005 Development of the working memory battery and its validity in primary school students: *no pdf available online*
17. Lu T. 2017 Validity and reliability of the Behavior Rating Scale of Executive Function-Preschool Version parent form in China: *no pdf available online*
18. Ebrahimi A. 2016 Psychometric properties of the Behavior Rating Inventory for Executive Function-Preschool (BRIEF-P) among preschool children: *only Persian version of pdf available*
19. Andrea K. 2010 The executive functions from a neuropsychometric perspective: study from Hungary, but *no pdf available online*
20. de Oliveira A. 2014 Construction of a Scale to Assess Cognitive Planning: *study conducted in Brazil but only Portuguese pdf version available *
21. y Vila Molina G. 2010 Taylor's Figure Standardization on Mexican population: *study conducted in Mexico but only Spanish pdf version available *
22. Musso M. 2009 Assessment of executive functions in children: Analysis and adaptation of tasks in a school context: *study conducted in Argentina but only Spanish pdf version available *
23. Qian Y. 2009 Reliability and validity of the Behavior Rating Inventory of Executive Function Teacher Form for school age children in China: *no pdf available*
24. Butman J. 2000 Spanish verbal fluency test. Normative data in Argentina: *only Spanish pdf version available *
25. Abedi A. 2012 Standardization of the neuropsychological test of NEPSY on 3-4 years old children: *only Persian version of pdf available*
26. Ramos-Galarza C. 2019 EFECO scale for assessing executive functions in self-report format: *only Spanish pdf version available *

### Appendix III:

**Table 10:** Study Characteristics of All Critically Appraised Papers (Kusi-Mensah et al., 2021)

**Instrument Reference (authors, year)**	**Name and version of instrument**	**Outcome measure assessed**	**Country/ Study sites**	**Type of Study*: development, validation**	**Target Population**	**Mode of Admin**	**Sub-scales/ number of items of interest**	**Sample size**	**Age range and mean**	**Local Setting**	**Condition (clinical/ healthy)**	**Language(s) of population**
(Taşcu et al., 2012)	Wisconsin Card Sorting Test WCST	EF	Romania	construct validation (discriminant)	Mostly children and few adults	interview-based task	single task	1101	26.7	urban	clinical and healthy controls	Romanian
(Amini et al., 2016)	Iranian Children's Participation Assessment Scale (CPAS)	Adaptive functioning	Iran	Scale development	Children	parent report/self-report	8 subscales: ADL, Instrumental ADL, Play, Leisure, Social Participation, education, Work, Rest/Sleep	40 kids, 21 parents	11.5 (2.8); range 6–18 years	urban	healthy	Persian
(Ford et al., 2019)	Rapid Assessment of Cognitive and Emotional Regulation (RACER)	EF	Lebanon, Niger	cross cultural validation	children	interview-based, computer administered task	2: inhibition; working memory	2725; 866–Niger, 1859 Lebanon	Lebanon: 9.2 (2.3); Niger: 9.2 (1.4)	urban	healthy	French; Arabic
(Malkawi et al., 2015)	Arabic Preschool Activity Card Sort (A-PACS)	adaptive functioning, ADL	Jordan	development	school-age children	parent report	7 domains: self-care, community mobility, high physical demand leisure and low physical demand leisure, social inter- action, domestic and education	115 caregivers	4.8	rural and urban	healthy	Arabic
(Senturk et al., 2014)	Junior Brixton Test (JBT)	EF	Turkey	Internal structure (structural validity), construct validation (convergent)	School-age children	interview-based task	single task	121	7.2	urban	healthy	Turkish
(Sobeh & Spijkers, 2012)	KITAP	Attention, inhibition, cognitive flexibility	Syria	Cross cultural validation, Construct validation (discriminant)	school-age children	performance-based task	Flexibility (The Dragon's House), Go/No-Go subscales	143	8	urban	healthy	Syrian Arabic
(Rosete, 2018)	Delis Kaplan Executive Function System (DKEFS); Children’s Color Trails Test (CCTT); Color Trails Test (CTT)	EF	Thailand	Structural validation	adolescents	interview-based tasks	Design Fluency and Verbal fluency subscales of DKEFS	156	16.44 (2.04)	urban	healthy	Thai
(de Bustamante Carim et al., 2012)	Behavior Rating Inventory of Executive Function (BRIEF)	EF	Brazil	structural validity and internal consistency	children and adolescents	self-report, parent and proxy (teacher) report	8 sub-scales: inhibition, flexibility and emotional control (behavioural regulation index), initiative, working memory, planning / organization, material organization and monitoring (metacognition index)	277 parents; 282 teachers; 112 adolescents	9.8 (3.4)	urban	healthy	Portuguese
(Korzeniowski & Ison, 2019)	Executive Function Scale for Children EFS	EF	Argentina	content validation, structural validity, internal consistency, reliability (test–retest)	school-age children	parent report	6 sub-scales: attention control, inhibitory control, metacognition, organization, planning and cognitive flexibility	307	7.7 (1.07)	urban	healthy	Spanish
(Burkey et al., 2015)	BRIEF	EF	Uganda	Internal consistency	school-age children	parent report	8 sub-scales: as above for BRIEF	185 children: 28 ADHD, 157 healthy	8.5 years	urban	clinical (ADHD) and healthy	Luganda
(Kashala et al., 2005)	Design Copying, Tower Test (from NEPSY), Digit Span (backwards)	EF: planning, working memory	DR Congo	Structural validation, construct validation (discriminant)	children	interview-based task	Design Copying, Tower Test (from NEPSY), Digit Span (backwards)	185 (28 cases, 157 controls)	7–9.9 years, mean 8.5 years	urban	mixed: 15% clinical (ADHD), 85% healthy	French; assorted African dialects
(Holding et al., 2018)	Rey-Osterrieth complex figure (ROCF), Go/No-Go, Shift	EF: Working Memory, Inhibition, cognitive flexibility	Bangladesh, Ghana, Tanzania	adaptation, internal consistency, cross cultural validation, reliability (test–retest, inter rater), responsiveness	children	interview-based task	3 scales: each 1 item/task given multiple times	786 total: 166 Ghana, 323 Tanzania, 297 Bangladesh	range: 7–18; mean: 13	rural	healthy	Kasem and Nankam (Ghana), Swahili (Tanzania), Chatagonian (Bangladesh)
(Willoughby et al., 2019)	EF Touch (computer-based battery)	EF: inhibition, cognitive flexibility and working memory	Kenya	construct validation (discriminant)	pre-school children	computer-based, interview administered task	5 tasks/subscales: Go/no-go, variant of Stroop, Spatial conflict arrows, Pick the picture, Something's the same	193	range: 3–6yrs	urban	healthy	Kiswahili, English
(Bakar et al., 2011)	BRIEF	EF	Turkey	construct validation (convergent and discriminative)	school-age children	parent report	8 sub-scales: as above for BRIEF	80: 61 ADHD, 19 controls	6–11 years;	urban	ADHD and healthy	Turkish
(Garcia-Barrera et al., 2015)	Behavioural Assessment System for Children (BASC)	EF	Colombia	internal consistency, cross cultural validation, Construct validation (discriminative)	school-age children	parent report	Not stated	848 healthy; 155 clinical	6–11 years	urban	ADHD and healthy	Spanish
(Rosetti et al., 2018)	Ball Search Field Task (BSFT), BRIEF	EF	Mexico	construct validation (convergent and discriminative (age)), internal consistency (for BRIEF)	children and adolescents	interview-based task; parent report (BRIEF)	single task	106	range: 6–16; mean: 10.2 (2.7)	urban	ADHD only	Spanish
(Tombokan-Runtukahu & Nitko, 1992)	Indonesian- Vineland Adaptive Behaviour Scales (VABS)	adaptive functioning	Indonesia	adaptation (development), internal consistency, reliability (intra-rater and inter rater), cross cultural validation, Construct validation (discriminant)	children	parent report	4 domains: Communication, Daily Living Skills, Socialization, and Maladaptive Behavior	86: 43 ID, 43 healthy	range: 6- 18; mean: 11.8 (2.9)	semi-urban	Intellectual disability, healthy	Indonesian
(Amini et al., 2016)	Iranian- Child Participation Questionnaire (I-CPQ)	adaptive functioning	Iran	Structural validity, internal consistency, reliability, construct validity (convergent)	pre-school children	parent report	6 subscales: self-care, home participation, play, leisure, social participation, and educational environment	120	range: 4–6; mean: 5.2	urban	cerebral palsy	Persian
(Malek et al., 2013)	Stroop Colour Word Test (Victoria Edition)	EF: inhibition	Iran	construct validation (discriminative), reliability (test–retest)	adolescents	interview-based task	single task	180: 150 healthy, 30 ADHD	range: 12–17 years	urban	ADHD and healthy	Persian and Turkish
(Sallum et al., 2017)	Self-Ordered Pointing Task (SOPT)	EF: working Memory	Brazil	construct validation (discriminative and convergent), ecological validity	pre-school children	interview-based task	single task	248	range: 3–5	urban	healthy	Portuguese
(Zarrabi et al., 2015)	BRIEF	EF	Iran	construct validation (discriminative)	school-age children	parent report	8 sub-scales: as above for BRIEF	60: 30 cases, 30 controls	range: 7–12 years; mean: 9yrs (1.41)	urban	ADHD and healthy	Persian
(Holding & Kitsao-Wekulo, 2009)	Participation in Activities of Daily Living (PADL)	adaptive functioning	Kenya	development, internal consistency, construct validity (discriminative)	children	interview-based questionnaire	2 subscales: participation and limitations to participation	study development: 92, validation: 116	range: 6–18; mean: 11.7 (2.9)	rural	assorted chronic conditions (Sickle Cell, DM, etc.); acute encephalopathy; healthy controls	Kiswahili
(Mashhadi et al., 2020)	Barkley Deficits in Executive Functioning Scale–Children and Adolescents (BDEFS-CA)	EF	Iran	structural validation, internal consistency, reliability (test–retest), convergent validity, discriminative validity	children and adolescents	parent report/self report	5 subscales: Self- Management to Time, Self-Organization/Problem Solving, Self-Restraint, Self-Motivation and Self-Regulation of Emotion	2295	range: 6–18; mean: 11.6 (3.34)	rural and urban	mostly healthy, 4.7% clinical	Persian
(Tol et al., 2011)	Child Function Impairment Rating Scale (CFIRS)	adaptive functioning	Indonesia	development, Structural Validity, internal consistency, reliability (test- retest, inter rater), construct validity (convergent and discriminant)	children	FGD, diaries, interview-administered questionnaire	1 unidimensional scale with 4 factors; 11 items,	development: 53, validation: 403 children, 385 parents	mean: 9.9 (1.21)	rural	healthy but exposed to political violence	Indonesian
(Sartori et al., 2020)	Go/No-go app version	EF: inhibition	Brazil	content, structural validity, internal consistency, cross-cultural, and construct (convergent and discriminant)	school-age children	interview-based task	4 tasks of inhibition	306: 253 healthy, 53 DCD	range: 8–10 years	urban	Development Coordination Disorder, healthy	Portuguese
(Chernoff et al., 2018)	BRIEF	EF	South Africa, Malawi, Zimbabwe, Uganda	adaptation, reliability (test–retest), measurement error, convergent validity	school-age children	parent report	8 sub-scales: as above for BRIEF	603: 244 HIV, 179 HIV-exposed,180 no HIV)	range: 5–10	rural and urban	HIV, HIV exposed but healthy, no HIV exposure	Luganda, Afrikaans, Xhosa, Shona, Zulu, Chichewa, Sesotho, and Setswana
(Nampijja et al., 2010)	WCST, Knock-Tap Game (Go/No-Go)	EF: cognitive flexibility, planning	Uganda	adaptation, reliability (test–retest), discriminative validity	pre-school children	interview-based task	single task (each)	64	range: 4–6; mean: 5.2	rural and urban	healthy	Luganda
(Thorell et al., 2020)	Teenage Executive Functioning Inventory (TEXI)	EF: Working memory, inhibition	Serbia	adaptation, structural validation, internal consistency, reliability (inter-rater)	adolescents	self-report, parent and proxy (teacher) report	20 items, 2 subscales: inhibition and working memory	302 adolescents	range: 13–19; mean 16.4 (1.8)	rural and urban	healthy	Serbian
(Barreto et al., 2018)	Neuropsychological Assessment of Executive Functions (ENFEN)	EF	Colombia	structural validation, construct validation (convergent and discriminant)	school-age children	interview-based task	Not stated	367 children	range: 6–12; mean 8.9 (1.8)	rural and urban	healthy	Spanish
(Yang et al., 2018)	Dysexecutive Questionnaire (DEX)	EF	China	structural validation	adolescents	self-report	20 items	1586	mean: 18.9 (5.4)	urban	healthy	Mandarin
(Munir et al., 1999)	Independent Behaviour Assessment Scale (IBAS)	adaptive functioning	Bangladesh	reliability, internal consistency, construct validity (discriminant)	pre-school and school-age children	parent report, interview-based tasks	4 subscales: Motor skills, Socialisation skills, Communication skills, Daily living skills	1404 healthy, 222 clinical	range: 2–9,	rural and urban	healthy and clinical	Bengali
(Selvam et al., 2016)	VABS II- Indian Version	adaptive functioning	India	cross cultural validation	pre-school children	parent report	4 domains: as above for VABS	412	2–6 years,	urban	healthy	Kannada
(de Siqueira et al., 2016)	Child Hayling Test- Brazilian version	EF: inhibition, cognitive flexibility	Brazil	adaptation (development)	school-age children	interview-based task	24 items	139	range: 6–12	urban	healthy	Portuguese
(Richard’s et al., 2017b)	Conjunction Visual Search (CVS) task of tareas de autorregulaCión Cognitiva (TAC) battery	EF: inhibition	Argentina	development (but of computer-based task), convergent validity	school-age children	interview-based, computer administered task	1 task: 120 trials	41	range: 6–11, M 8.49; SD 1.47	urban	healthy	Spanish
(Injoque-Ricle et al., 2011)	The Automated Working Memory Assessment (AWMA)	EF: working Memory	Argentina	adaptation (development), internal consistency, construct validity (convergent)	school-age children	interview-based task	12 tasks: Digit Recall, Word Recall, Non-word Recall, Dot Matrix, Block Recall, Mazes Memory, Listening Recall, Counting Recall, Backward Digit Recall, Odd One Out, Mr. X Spatial Span	26 adaptation; 210 validation	range 6–11	urban	healthy	Spanish
(Selvam et al., 2018)	BRIEF- pre-school version	EF	India	structural validity, internal consistency, reliability (inter rater), cross cultural validation, discriminant validation	pre-school children	parent report	5 sub-scales: inhibition, change, emotional control, working memory and planning / organization	412	2–5 years	urban	healthy	Kannada
(Amani et al., 2018)	BRIEF- teacher	EF	Iran	structural validation, internal consistency, construct validity (convergence)	school-age children	proxy (teacher) report	8 sub-scales: as above for BRIEF	360 children, 12 teachers	range: NR	urban	healthy	Persian
(Amini et al., 2017)	CPAS- parent version	adaptive functioning	Iran	structural validation, reliability (test–retest), construct validation (convergent)	school-age children	parent- report	8 subscales: ADL, Instrumental ADL, Play, Leisure, Social Participation, education, Work, Rest/Sleep	700 parents	range: 6–12; mean 9.45 (1.76)	urban	healthy	Persian
(Green et al., 2019)	CANTAB	EF: working memory, inhibition, and attention shifting	Mexico	construct validation (discriminative)	children and adolescents	interview-based task, computer based	6 sub-tests (out of 23): stockings of Cambridge, delayed matching sample, extra dimensional shift, rapid visual information processing, stop signal task and match to sample visual search task	826 children	range 5–15; mean 9.27 (2.6)	urban	healthy	Spanish
(Richard’s et al., 2018)	Tareas de Autorregulación Cognitiva Battery (TAC)	EF: inhibition, working memory and cognitive flexibility	Argentina	structural validity	children	interview-based task	3 sub-scales used: perceptual inhibition task, 4 WM tasks; 1 cognitive flexibility task	103 children	range: 9–12 years; M = 10.84 SD = 0.88	urban	healthy	Spanish
(Du et al., 2018)	Symptoms and Functional Impairment Rating Scale (SFIRS)	EF in ADHD: working memory, planning, time management, self-monitoring, and emotional control	China	development, structural validity, internal consistency, reliability (test- retest), construct validity	school-age children	parent report	6 sub-scales: hyperactivity-impulsivity, self-control, inattention, self-management, school performance, social interaction	20 development; 412 validation	range: 6–12, 8.7 (1.42)	urban	ADHD and healthy	Mandarin
(Pineda et al., 2007)	bespoke battery including: WCST- abbreviated, verbal fluency test, token test, ROCF	EF in ADHD	Colombia	structural validation, discriminant validation	school-age children	interview-based task	3 tasks	621 total: 249 cases, 372 controls	range: 6–11,	urban	ADHD and healthy	Spanish
(Trevisan et al., 2017)	Children’s Executive Function Inventory (CHEXI)	EF in ADHD	Brazil	adaptation (development), structural validation, internal consistency, construct validity (convergence or concurrent)	pre-school children	parent report/ proxy report	4 subscales: working memory, inhibition, self-regulation and planning	408 parents and teachers	range 4–7, M = 5.51, SD = 0.59	urban	ADHD and healthy controls	Portuguese
(Arruda et al., 2020)	EFICA (Executive Function Inventory for Children and Adolescents)- parent and teacher versions	EF	Brazil	structural validation, internal consistency, reliability, construct validity (discriminant)	children and adolescents	parent report/ proxy (teacher) report	Not stated	3284 healthy, 165 ADHD	range 5–18, mean 8.2 (2.0)	rural and urban	ADHD and healthy	Portuguese
(Pluck et al., 2019)	Tower Test of DKEFS	EF: planning, working memory	Ecuador	Structural validity, internal consistency, reliability (test- retest)	children and adolescents	interview-based task	1 task	264	range 10–20, mean 15.05	urban	healthy, but some street children and foster children with controls	Spanish
(Wong et al., 2012)	Ballet Executive Scale (BES)	EF in dance/ballet	Cuba	structural validation, internal consistency, concurrent validity	adolescents	self-report	5 subscales: Strategic Planning, Organization of Dance Behavior, Motivation to Dance, Impulse Control in Dance Behavior, Empathy toward Other Dancers	149	range: 11–18	urban	healthy	Spanish
(Diaz & Anacona, 2017)	BRIEF- pre-school version	EF	Colombia	adaptation, structural validation, internal consistency, test–retest reliability, construct validation (convergent)	pre-school children	parent report	5 sub-scales: inhibition, change, emotional control, working memory and planning / organization	125	range: 2–5 years; mean 4.3	urban and rural	healthy	Spanish
(Goldberg et al., 2009)	VABS- Vietnam version	adaptive functioning	Vietnam	adaptation, structural validity, internal consistency, construct validation (discriminant)	pre-school children	parent report	4 domains: as above for VABS	120 healthy, 31 ID children	range: 3–6yrs; mean 4.9 (1.1)	urban	intellectual disability and healthy	Vietnamese
(Ruffieux et al., 2010)	Assessment Battery containing: Color Trail Test, Hand movements, Verbal Fluency, letter numbering sequence	EF in Sickle Cell Disease	Cameroon	Structural validity, inter rater reliability	children and adolescents	interview-based task	14 tasks: but only 5 relevant for EF's	125	range: 6–20, mean 11.4 (4.2)	urban	healthy	French, unnamed local dialects
(Gonen et al., 2019)	Preschool Self-Regulation Assessment. (PRSA)	EF in pre-schoolers: self-regulation	Turkey, USA	cross cultural validation, reliability	pre-school children	interview-based task	3 tasks: The Balance Beam, Tower Task and Pencil Tap tasks	Turkey: 471 children + caregivers; USA: 286 children + caregivers	Turkey: 5.23yrs (2.83 to 5.92); US 4.12 yrs (2.97 to 5.07)	urban	healthy	Turkish, English
(Xu et al., 2020)	computerized version of: Stop Signal task; Figure Matching task, Spatial Span task; Tower of Hanoi task	EF	China, Hong Kong and USA	cross cultural validation	school-age children and adolescents	self-administered computer-based tasks	4 tasks each measuring: inhibition, cognitive flexibility, working memory, planning	China: 453; Hong Kong 371; UK 487	HK: 12.2yrs, UK: 11.9yrs; China: 11.93yrs	urban and rural	healthy	Mandarin, Cantonese, English

## Footnotes

1 ICD 11 is due to be released on Jan 1^st^, 2022 hence still using ICD 10 nomenclature.
