# Correction to: A Systematic Review of the Validity and Reliability of Assessment Tools for Executive Function and Adaptive Function Following Brain Pathology among Children and Adolescents in Low- and Middle-Income Countries

**DOI:** 10.1007/s11065-022-09542-7

**Published:** 2022-04-30

**Authors:** Kwabena Kusi-Mensah, Nana Dansoah Nuamah, Stephen Wemakor, Joel Agorinya, Ramata Seidu, Charles Martyn-Dickens, Andrew Bateman

**Affiliations:** 1 Department of Psychiatry, University of Cambridge, Clifford Allbutt Building, Cambridge Biomedical Campus CB2 OAH, Cambridge, UK; 2 Komfo Anokye Teaching Hospital, P. O. Box 1934, Kumasi, Ghana; 3 Pantang Hospital, Accra, Ghana; 4 Department of Psychiatry, University of Michigan Health System, 1500 E Medical Center Dr, Ann Arbor, 48109 MI USA; 5 Accra Psychiatric Hospital, Accra, Ghana; 6 School of Health and Social Care, University of Essex, Colchester, UK

## Correction to: Neuropsychology Review https://doi.org/10.1007/s11065-022-09538-3

The original version of this article unfortunately contained mistakes.

1. There is a spelling error in the article title. "Reliability" has been misspelt as "Realiability".

2. The affiliation of Stephen Wemakor has remained the old institution of Komfo Anokye Teaching Hospital. His current institutional affiliation is: Department of Psychiatry, University of Michigan Health System, 1500 E Medical Center Dr, Ann Arbor, 48,109 MI, USA.

The original article has been corrected.

